# Assessing the impact of Australia’s mass vaccination campaigns over the Delta and Omicron outbreaks

**DOI:** 10.1371/journal.pone.0299844

**Published:** 2024-04-16

**Authors:** Lixin Lin, Haydar Demirhan, Simon P. Johnstone-Robertson, Rajiv Lal, James M. Trauer, Lewi Stone

**Affiliations:** 1 Mathematical Sciences, School of Science, RMIT University, Melbourne, Australia; 2 School of Public Health and Preventive Medicine, Monash University, Melbourne, Australia; 3 Faculty of Life Sciences, Biomathematics Unit, School of Zoology, Tel Aviv University, Tel Aviv, Israel; Centers for Disease Control and Prevention, UNITED STATES

## Abstract

**Background:**

The Australian Government implemented a national vaccination campaign against COVID-19 beginning February 22, 2021. The roll-out was criticised for being delayed relative to many high-income countries, but high levels of vaccination coverage were belatedly achieved. The large-scale Omicron outbreak in January 2022 resulted in a massive number of cases and deaths, although mortality would have been far higher if not for vigorous efforts to rapidly vaccinate the entire population. The impact of the vaccination coverage was assessed over this extended period.

**Methods:**

We considered NSW, as the Australian jurisdiction with the highest quality data for our purposes and which still reflected the national experience. Weekly death rates were derived among individuals aged 50^+^ with respect to vaccine status between August 8, 2021 and July 9, 2022. We evaluated deaths averted by the vaccination campaign by modelling alternative counterfactual scenarios based on a simple data-driven modelling methodology presented by Jia et al. (2023).

**Findings:**

Unvaccinated individuals had a 7.7-fold greater mortality rate than those who were fully vaccinated among people aged 50^+^, which rose to 11.2-fold in those who had received a booster dose. If NSW had fully vaccinated its ~2.9 million 50^+^ residents earlier (by July 28, 2021), only 440 of the total 3,495 observed 50^+^ deaths would have been averted. Up to July 9, 2022, the booster campaign prevented 1,860 deaths. In the absence of a vaccination campaign, ~21,250 COVID-19 50^+^ deaths (conservative estimate) could have been expected in NSW i.e., some 6 times the actual total. We also find the methodology of Jia et al. (2023) can sometimes significantly underestimate that actual number.

**Interpretation:**

The Australian vaccination campaign was successful in reducing mortality over 2022, relative to alternative hypothetical vaccination scenarios. The success was attributable to the Australian public’s high levels of engagement with vaccination in the face of new SARS-COV-2 variants, and because high levels of vaccination coverage (full and booster) were achieved in the period shortly before the major Omicron outbreak of 2022.

## Introduction

**Fig 1:**
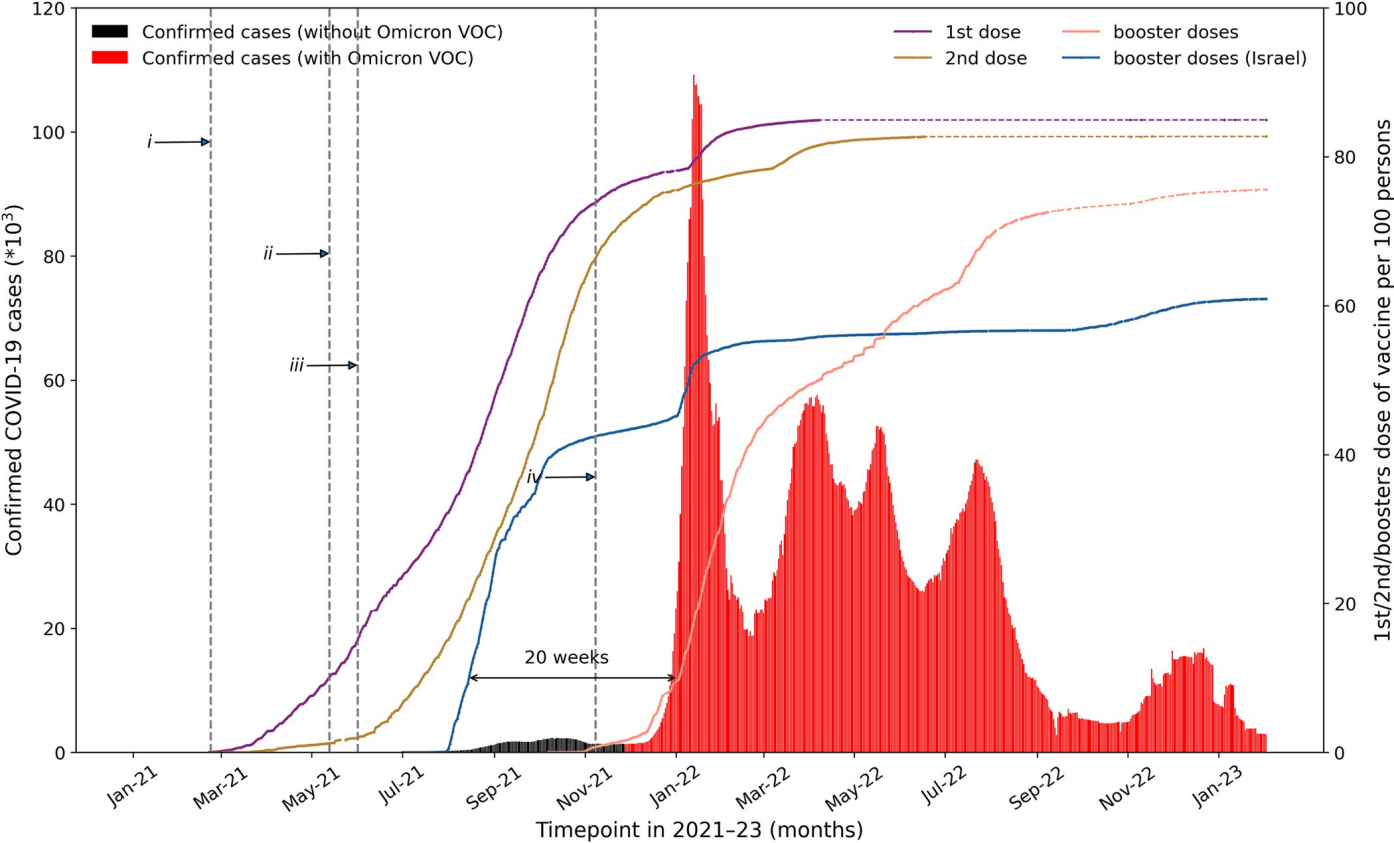
Epidemiological COVID-19 timeline in Australia. Confirmed cases [5] (7-day rolling average) of SARS-CoV-2 for all ages from 1 January 2021 to 2 February 2023 for the whole Australian population (left vertical axis). Red shading distinguishes the Omicron variant of concern from other strains (black). Solid lines [5] indicate cumulative vaccination coverage of first vaccination dose (purple), second dose (olive) and third “booster” (light red). Booster coverage in Israel (blue line; vertical axis on the right). Grey dashed lines [6,7] illustrate the Australian vaccination policy timeline: *i*) National public vaccination campaign begins on 22 February 2021. *ii*) 50^+^ were eligible to receive a COVID-19 vaccine from 3 May 2021. *iii*) 40^+^ were eligible to receive a COVID-19 vaccine from 8 June 2021. *iv*) Booster program initiated for 18^+^ on 8 November 2021.

Before June 2021, the use of non-pharmaceutical interventions such as lockdowns, border closures, travel restrictions, social distancing and enhanced case detection and tracing were the mainstays of outbreak control in the absence of available drugs or vaccines in Australia [1–3]. In NSW, by the end of May 2021, fewer than 60 people had died with COVID-19, and there had been almost no community transmission [4]. However, in mid-June 2021 the Delta variant of SARS-CoV-2 (lineage B.1.617.2) arrived in NSW and initiated an outbreak, with a peak of confirmed cases in October 2021 (Fig 1). On 28 November 2021 [4], the Omicron variant (lineage BA.1) arrived in NSW giving rise to the huge spike of infections in January and February 2022 and which continued on at lower intensity over the year (Fig 1). A question of great interest is: What are the factors that contributed to the initiation and rapid decline of this major spike? The same patterns are qualitatively similar to those observed in most other States in Australia (Fig A in S1 File). More specifically, what is the relationship between the vaccination campaign and the evolution of the epidemic? And is it possible to estimate the total deaths averted by the vaccination campaign?

To proceed, it is important to consider vaccination coverage. Australia was relatively late in mass vaccinating its population during the SARS-CoV-2 pandemic, beginning its program on 22 February 2021 [6]. Four months later, less than 5% of the population were fully vaccinated with two doses, making it the second-lowest vaccination coverage of any OECD nation at the time (Fig D in S1 File). Yet a few months later at the end of 2021, Australia had achieved one of the highest vaccination rates in the world, [8] with more than 85% of its eligible population fully vaccinated with at least two doses. Due to the rapid pace of the Australian roll-out in the latter half of 2021 (Fig 1) and the very high public engagement with the program, vaccination coverage was at high levels when Omicron arrived. On the one hand, the delayed start left a large immune naive population dangerously vulnerable to any newly emerging SARS-CoV-2 Variants of Concern (eg., Delta and Omicron). But on the other, when Australia’s Omicron outbreak began in November 2021, the delay of the vaccination program was actually advantageous since it limited the extent of immunity waning, and so increased protection during the Omicron period. Fortuitously, Australia’s slow start should, in hindsight, not necessarily be viewed as detrimental.

Based on the limited data available on deaths and vaccination coverage, and using a data-driven modelling approach similar to recent studies in the literature [9–12], in this paper, we sought to answer questions evaluating the success or failure of the Australian vaccination program: How many deaths could have been averted if the vaccination program had been rolled out more rapidly, such that the population was fully vaccinated within 6 months by 28 July 2021. How many deaths were averted by the booster vaccine? How many deaths would have occurred in the absence of vaccination? How was the unvaccinated population affected through the pandemic, especially in comparison to the vaccinated? While it’s challenging to provide precise quantification for the above questions due to the limited available data, even conservative estimates of these quantities have proven to be both interesting and valuable.

The data-driven modelling approach used in our paper may appear less sophisticated than dynamical SIR modelling approaches, but it successfully provides an overview guide of basic epidemiological trends and processes that occur during the vaccination period. As such, it may also have some advantages, especially given that data availability is limited and a number of the many SIR parameters would be difficult to estimate. Apart from our work, only limited assessments have been attempted in Australia because of limited data. Related approaches have been used in landmark studies in the USA [11], Israel [10] and Japan [13]. These methodologies have been extended here in several ways, including consideration of waning immunity.

## Materials and methods

### Data

Our analysis draws from datasets made publicly available by the Health Ministries of State and Federal governments in Australia. Access to full official Australian government datasets was not possible despite extensive efforts. As such, we concentrated on data from the state of New South Wales (NSW) with a population of 8.1 million (Australia’s largest state, with 31.8% of the national population). As Australia’s COVID-19 outbreaks were geographically relatively homogeneous (apart from Western Australia), conclusions from the NSW datasets should be representative of the nation.

**Fig 2:**
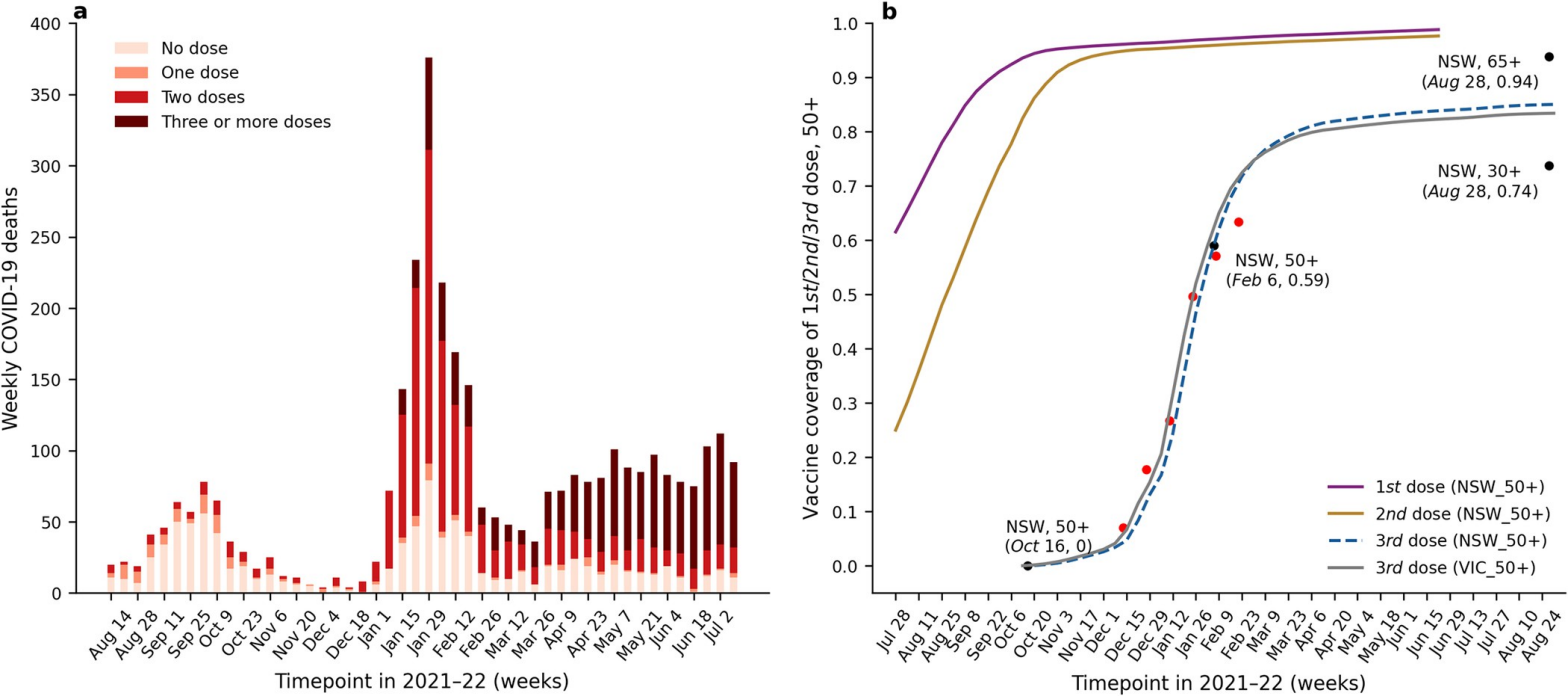
Weekly COVID-19 deaths and vaccination coverage. Panel a: NSW: Processed weekly COVID-19 deaths by vaccination status, aged 50^+^ in NSW over the study period from 8 August 2021 to 9 July 2022, with a total of 3,495 deaths. Data obtained from weekly reports published by the NSW Ministry of Health [4,14]. Tick is week ending date. Panel b: Vaccination coverage of people aged 50^+^ in Victoria and NSW. Third dose coverage in NSW (blue dashed line) is taken to be similar to VIC (grey line) but shifted 5-days earlier. The estimate is corroborated by other points (red, black; details in Part B of the S1 File). Tick is exact date.

Our primary focus was on individuals aged 50 and above (50^+^), as the majority of COVID-19-related deaths occur in this age bracket, and finer resolution age-class data for NSW (or any other state) were either unavailable or insufficient. As found in publicly available weekly reports published by the NSW Ministry of Health [4,14], we recorded the 3,495 weekly COVID-19 confirmed deaths in NSW for the 50^+^ population (Fig 2A), with a total population of *N* = 2,885,951 [15]. These data cover a 48-week period from weeks *t* = 1 (14 August 2021, week ending date) to *t* = 48 (9 July 2022), and are classified by vaccination history as either having received no doses, one dose, two doses (fully vaccinated), or three or more doses.

Publicly available COVID-19 vaccination data (Fig 2B) were obtained from the Australian Government’s Department of Health and Aged Care [16]. The national public vaccination campaign began on 22 February 2021, but by 2 July 2021, only 10% [17] of the NSW population aged 50^+^ had received two doses of the vaccine (i.e., full vaccination). The booster campaign was also delayed and did not begin until November 8, 2021, almost 20 weeks later than in Israel (Fig 1). Nevertheless, by early January 2022, almost 95% of the NSW population aged 50^+^ had received two doses of the vaccine. In addition, by early February 2022, almost 60% of the NSW population aged 50^+^ had received a third booster dose of the vaccine.

Additional details regarding COVID-19 death data and vaccination data can be found in Parts A and B of the S1 File, including definitions related to COVID-19 deaths and vaccination status in Part D of the S1 File.

### The data-driven model

The rates of mortality of 50^+^ individuals were first estimated across four vaccination status categories, in any single week. These were then used to estimate the mortality timeseries if the vaccination dose schedule had been changed from that observed in reality. The primary model input was the number of COVID-19 deaths with no dose (i.e., unvaccinated), one dose, two doses and three or more doses in week *t*, which we denote *d*_*u*_(t), *d*_1_(*t*), *d*_2_(*t*), and *d*_3+_(*t*) respectively. To allow for delays in reporting, in a separate analysis the mortality data was shifted earlier with one week delay, but this changed results minimally (Table D in S1 File). The proportion of the population (*N*) having only *k*-doses is *v*_*k*_(*t*) etc. In determining *v*_*k*_(*t*) from the data, we assumed that any vaccine dose required 2 weeks to provide protective immunity, which we implement by shifting vaccination coverage (1^st^ dose, 2^nd^ and 3^rd^ dose) two weeks later (Fig 2B). The proportion of the population unvaccinated in week *t* was denoted as *v*_*u*_(*t*) = [1‒*v*_1_(*t*)‒*v*_2_(*t*)‒*v*_3+_(*t*)]. Thus, the number of unvaccinated individuals in the full population is *v*_*u*_(*t*)*N*.

The death rate of unvaccinated individuals in week *t*, *r*_*u*_(*t*), was calculated as the number of COVID-19 unvaccinated deaths amongst all the unvaccinated in the population i.e., $r_{u}(t)=\frac{d_{u}(t)}{v_{u}(t)N}$. Extending this, we let *r*_*k*_(*t*) represent the vaccination status-specific (VS) death rate for individuals who received only*k*-doses, and is given by: $r_{1}(t)=\frac{d_{1}(t)}{v_{1}(t)N},\;r_{2}(t)=\frac{d_{2}(t)}{v_{2}(t)N},\;r_{3+}(t)=\frac{d_{3+}(t)}{v_{3+}(t)N}$. The cumulative number of deaths amongst individuals with *k*-doses by the end of the study period is denoted by *d*_*k*_, where the dependence on *t* is dropped. According to the dataset, the D* = 3,495 deaths among 50^+^ over the study period were comprised of: *d*_*u*_ = Σ_*t*_ *d*_*u*_ (*t*) = 959, *d*_1_ = Σ_*t*_ *d*_1_ (*t*) = 67, *d*_2_ = Σ_*t*_ *d*_2_ (*t*) = 1295, and *d*_3+_ = Σ_*t*_ *d*_3+_ (*t*) = 1074. That is, *d*_*u*_ = 959 unvaccinated aged 50^+^ individuals had COVID-19 related deaths over the study period, etc. Counterfactual cumulative COVID-19 deaths were then calculated under three broad “what if” scenarios. See Part C of the S1 File for more details on each scenario.

#### Scenario I. How many deaths could have been averted if complete vaccination coverage had been achieved as early as 28 July 2021?

Jia et al. (2023) [11] estimated that at least 232,000 deaths could have been prevented among unvaccinated adults during the 15 months study period had they been vaccinated with at least a primary series. Similarly, we examined how many deaths could have been averted if the entire NSW 50^+^ population had been fully vaccinated as early as 28 July 2021. As detailed in Part C of the S1 File, the data-driven model calculates the cumulative counterfactual deaths *D*_1_ as:

$$D_{1}=\sum_{t=1}^{48}\left[{r_{2}(t)v_{u}(t)N+r_{2}(t)v_{1}(t)N+r}_{2}(t)v_{2}(t)N+r_{3+}(t)v_{3+}(t)N\right].$$ (1)

Part C of the S1 Text describes an improved method to calculate *D*_1_ that allows for waning immunity as well as uncertainty in the weekly death rates (i.e., 80% prediction intervals), and the results of these modifications are presented in Table 1. Fig G in S1 File compares the differences with and without additional waning immunity allowed for.

**Table 1:** Estimated counterfactual averted deaths with 80% prediction interval (PI) for each scenario, as described in Part C of the S1 File.

Type	Total estimated 50^+^ deaths (*D*_*s*_)	Change in 50^+^ deaths
Observed deaths	3,495	0
I. Early achievement of full vaccination coverage by July 28 2021.	3,060 [80% PI:2,630–3,490]	+440 [80% PI: 10–890] averted deaths
II. No booster campaign	5,350 [80% PI: 4,780–5,940]	1,860 [80% PI: 1,290–2,440] extra deaths
III. No vaccination program	21,250 [80% PI: 20,100–22,410]	17,760 [80% PI: 16,610–18,920] extra deaths

#### Scenario II. How many deaths were averted by the booster vaccine?

Similarly, we estimate how many lives would have been lost if Australia failed to provide the booster vaccination, such that no-one in the population was vaccinated with three or more doses. The cumulative counterfactual deaths *D*_2_ is then given by:

$$D_{2}=\sum_{t=1}^{48}\left[{r_{u}(t)v_{u}(t)N+r_{1}(t)v_{1}(t)N+r}_{2}(t)v_{2}(t)N+r_{2}(t)v_{3+}(t)N\right].$$ (2)

Similarly waning immunity can be allowed for as detailed in Part C of the S1 File.

#### Scenario III. How many deaths would have occurred in the absence of vaccination?

Of all the scenarios examined here this is the most difficult to estimate accurately as various assumptions that are difficult to check need to be made (and this would be true for all modelling approaches we are aware of). In this scenario the entire population remains unvaccinated. The rate of deaths for each week of the 48-week study period is taken to be the rate at which the unvaccinated die with COVID-19 on that week multiplied by the population size i.e., *r*_*u*_ (*t*)*N*. Summing over all weeks of the analysis period, gives an estimate of the total cumulative number of 50^+^ COVID-19-related deaths that would have occurred in the absence of vaccination over the study period, namely:

$$D_{3}=\sum_{t=1}^{48}[r_{u}(t)N].$$ (3)

Equivalently this could be interpreted as the number of lives saved by the vaccination program. In order to improve this simple method, we have incorporated a correction that allows for waning immunity and for the depletion of susceptibles over the ensuing epidemic based on a *DIS* model. Technical details can be found in Part C of the S1 File.

#### Averted deaths

In each of the above scenarios, the number of deaths that could have been averted, or equivalently the number of lives saved, was estimated from

$$D_{averted}=D^{*}-D_{s},$$ (4)

Where D* = 3,495 is the total number of COVID-19 deaths aged 50^+^ during the study period in NSW, and *D*_*s*_ is the total number of counterfactual deaths for a given scenario. A positive value indicates that deaths could have been avoided if the counterfactual scenario had been implemented instead of the NSW policy, while a negative value indicates the converse.

## Results

### Unvaccinated

**Fig 3:**
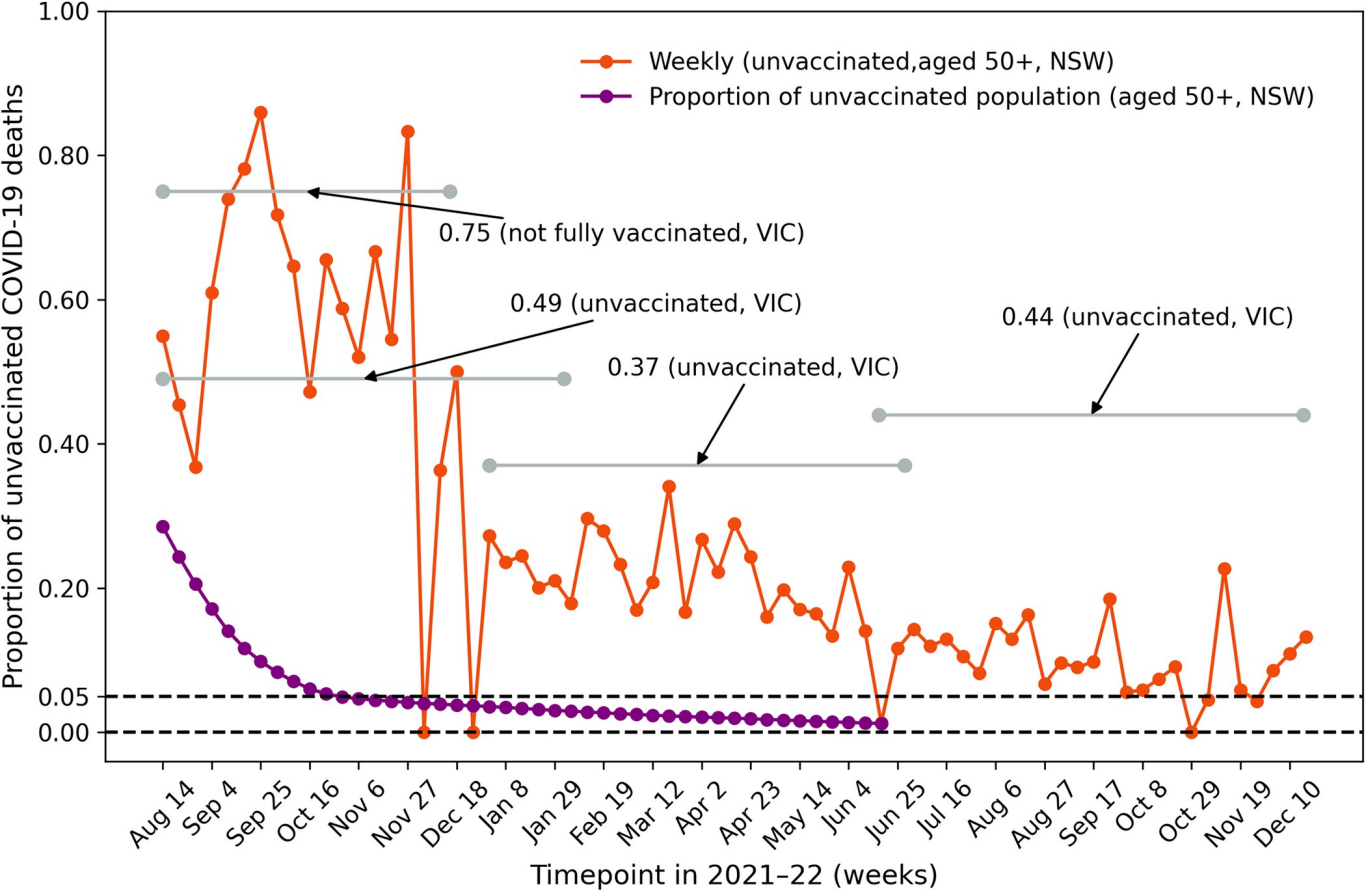
Proportion of unvaccinated COVID-19 deaths. Proportion of weekly COVID-19 50^+^ deaths who were unvaccinated in NSW (orange line); that is, *p*_*u*_(*t*) = *d*_*u*_(*t*)/*TD*(*t*). Data obtained from the NSW Ministry of Health [4,14]. Between 8 August 2021 and 17 December 2022, there were 5,397 COVID-19 50^+^ deaths, including 1,170 unvaccinated COVID-19 deaths. The proportion of unvaccinated amongst the deaths reached 86% during the Delta period in late September 2021. Four average values of the proportion are plotted for VIC, Australia’s second largest state (grey lines). The proportion of the 50^+^ population that is unvaccinated is plotted for NSW (purple line). Tick is exact date.

**Fig 4:**
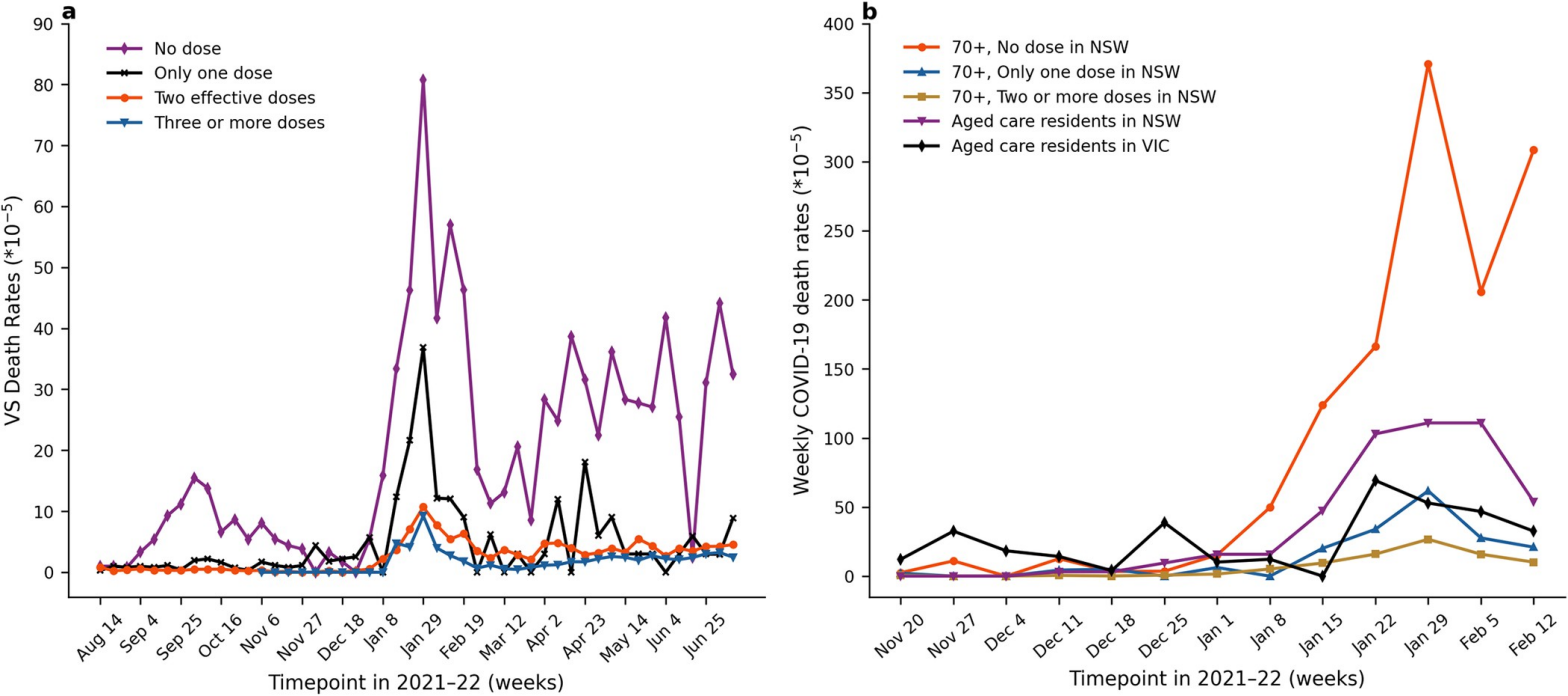
Weekly COVID-19 VS mortality rates. Panel a: The weekly COVID-19 VS mortality rates *r*_*k*_(*t*) in NSW individuals aged 50^+^ from 8 August 2021 to 9 July 2022. The no dose curve (purple) is *r*_*u*_(*t*), and represents the per capita rate at which members of the unvaccinated subpopulation died in week *t*. Panel b: Weekly COVID-19 VS mortality rates *r*_*k*_(*t*) in NSW individuals aged 70^+^ from 14 November 2021 to 12 February 2022, calculated using a similar method. Weekly mortality rates for NSW and VIC aged care residents (purple and black curves respectively) are shown for comparison, and for comparison with NSW 70+ data in general population. Tick is week ending date.

We first considered the degree to which unvaccinated individuals were over-represented in the mortality data. The proportion of unvaccinated 50^+^ COVID-19 deaths in week *t* is *p*_*u*_(*t*) = *d*_*u*_(*t*)/*TD*(*t*), where *TD*(*t*) = *d*_*u*_(*t*)+*d*_1_(*t*)+*d*_2_(*t*)+*d*_3+_(*t*) denotes the total COVID-19 deaths in week *t*. The proportion *p*_*u*_(*t*) is plotted as the epidemic evolved in time (Fig 3). During the Delta outbreak *p*_*u*_(*t*) was unusually large, reaching a maximum value of 86% in the week ending 25 September 2021. In contrast, less than 10% of the general (all ages) population was unvaccinated at the time, such that the unvaccinated subpopulation was extremely over-represented in the mortality data. We note a sharp decline or phase transition in *p*_*u*_(*t*) in early December 2021 when the Omicron outbreak began, largely due to Omicron’s immune evasion properties. From January to May 2022, *p*_*u*_(*t*) as on average 25% and dropped further down to 15–20% as 2022 progressed. Despite this, the unvaccinated population remained over-represented in mortality, given that *p*_*u*_(*t*) was almost always above 5% and *v*_*u*_(*t*), the proportion of unvaccinated in the 50^+^ population, was always below 5% during 2022 (see solid purple line). This fall in *p*_*u*_(*t*) from 86% to 15% attests to the higher transmissibility of Omicron compared to Delta [18]. The further gradual reduction over the latter half of 2022 in *p*_*u*_(*t*) may also partially be due to the depletion of the unvaccinated population as the number of vaccinations increased. Due to the limited availability of data, the proportion of unvaccinated 50^+^ deaths in Australia’s second-largest state of VIC could only be calculated for four periods (Fig 3; data from Victoria’s health department [19]), but showed broadly comparable results. Figs 3 and 4 clearly indicate the over-representation of the unvaccinated amongst the population who perished.

### Death rates

Fig 4A plots the NSW VS death rates *r*_*k*_(*t*), and is a particularly important figure that forms the basis for most of the modelling. The death rate amongst the unvaccinated *r*_*u*_(*t*) is highest, highlighting the increased vulnerability of this sub-population to COVID-19 mortality. The one-dose vaccination group also has a high VS death rate *r*_1_(*t*), especially in the first months of Omicron, and generally remains intermediate between the death rate in the unvaccinated and the doubly vaccinated. The unvaccinated had a 7.7 times higher average weekly death rate than those who were fully vaccinated among people aged 50^+^. Even more exaggerated, the unvaccinated had an 11.2 times higher average weekly death rate than those who had the booster among people aged 50^+^.

### Counterfactuals

**Fig 5:**
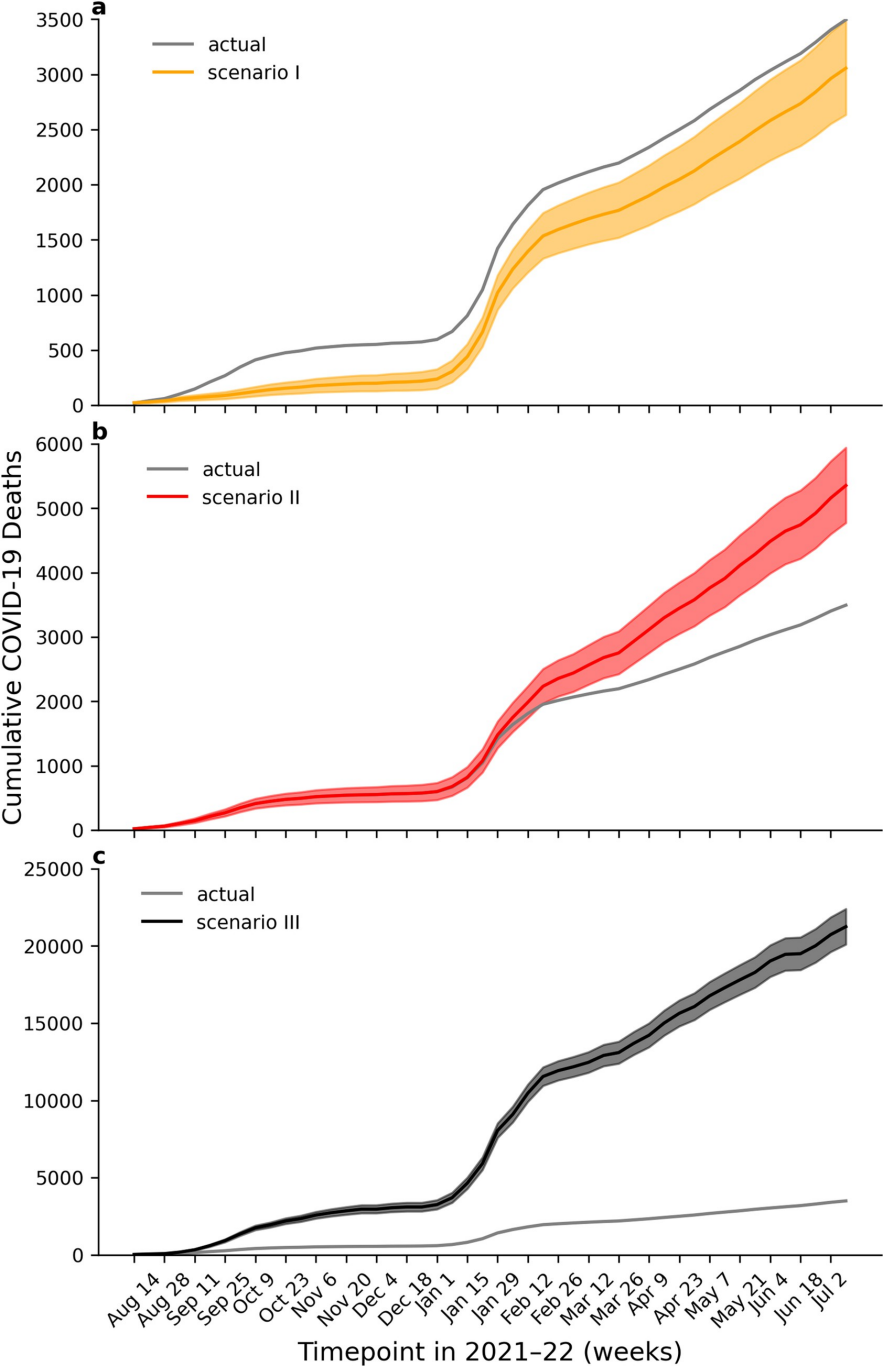
The cumulative number of counterfactual COVID-19 50^+^ deaths compared to the actual or observed number deaths (grey line) as a function of time in NSW. Panel a. Scenario I. Early achievement of high full vaccination coverage (orange solid). Panel b. Scenario II. No booster campaign (red solid). Panel c. Scenario III. No vaccination program (black solid). Shaded areas represent the 80% PI (Prediction Interval). Tick is exact date.

By applying the VS death rates shown in Fig 4A to different time-varying counterfactual proportions of the population, we explored the various “what if” scenarios described above (Methods). Table 1 (also Table C in S1 File) shows the total number of lives lost or deaths averted for each counterfactual scenario between 8 August 2021 and 9 July 2022, and Fig 5 shows the weekly variation.

#### Scenario I: Early achievement of high full vaccination coverage

If NSW had fully vaccinated its ~2.89 million 50^+^ residents earlier, specifically by the end of July 2021, only 440 [80% PI: 10–890] of the total 3,495 observed 50^+^ deaths would have been averted. This was calculated using a method that allows for the effects of waning immunity.

#### Scenario II: Absence of the booster

In the complete absence of a booster campaign, we estimated a further 1,860 [80% PI: 1,290–2,440] excess deaths in addition to the observed 3,495 COVID-19 50^+^ deaths. This should be viewed as a lower bound, and again allows for the effects of waning immunity.

### Scenario III: The absence of a vaccination campaign as a whole

In the absence of a vaccination campaign, an estimated 21,250 [80% PI: 20,100–22,410] COVID-19 50^+^ deaths would have been expected in NSW, which is approximately 6 times the observed total number. Again, this should be viewed as a conservative estimate, and again allows for the effects of waning immunity.

### Aged care residents

Data was available to compare the weekly COVID-19 death rates for NSW individuals aged 70^+^, as well as aged care residents from VIC and NSW, between 14 November 2021 and 12 February 2022. The death rates for each of these vulnerable populations is plotted in Fig 4B. Aged-care death rates in NSW (purple) were high, reaching some 50% of the death rate of the unvaccinated *r*_*u*_(*t*). In fact, the NSW aged care death rates were almost always worse than the one-dose death rates *r*_1_(*t*). The aged care 70^+^ death rates in the State of VIC were high but not as extreme as in NSW in 2022.

## Discussion

It is clear from Figs 2A and 3 that the great majority of Australia’s COVID-19 deaths were fully vaccinated individuals gives the false impression that vaccination was not necessarily beneficial. A more accurate picture is revealed by considering the VS death rate *r*_*u*_(*t*), i.e., the rate at which the unvaccinated population died with COVID-19. As shown in Fig 4A, the unvaccinated death rate is greater than that for any other vaccination status group, demonstrating that the unvaccinated are highly over-represented amongst COVID-19 deaths. This emphasises that the unvaccinated are the most unprotected and vulnerable group, correcting the misleading impression that could be gleaned from the raw data (Fig 2A).

### Scenario I

If Australia had rushed to full vaccination and achieved 100% coverage by the end of July 2021 (Scenario I), this would have almost completely mitigated the peak in deaths due to the Delta outbreak (Fig G in S1 File). The model indicates that 440 (mean) deaths or 13% of the observed total, would have been averted if waning immunity was reasonably accounted for. Some 360, or most of these deaths, would have been averted over the Delta wave up until December 2021. (However, if waning immunity is ignored, 930 deaths (27%; see Table C in S1 File) would have been averted by the end of the study period, which is approximately twice than that with the waning).

Jia et al. [11] used publicly available COVID-19-associated death rates to estimate the number of excess deaths that could be averted by vaccination in the US, with assumptions similar to our Scenario I, but did not adjust for waning immunity. Hence their conclusion "that at least 232,000 deaths [i.e., 50% of the total 457,000 reported COVID-19-associated deaths] could have been prevented among unvaccinated adults during the 15 months had they been vaccinated with at least a primary series" most likely overestimates the benefits of early full vaccination campaigns. Note that since >92% of the NSW 50^+^ population was in any case fully vaccinated by November 2021, an earlier vaccination campaign made little difference for the Omicron wave, at least until the end of the study period (Fig G in S1 File).

### Scenario II

In the complete absence of the booster vaccination, the model predicted an additional 1,860 deaths (i.e., 53% more than observed) over the study period, as a conservative estimate that also takes into account waning immunity. That is, a sum total of 5,350 COVID-19 deaths. It was expected that the booster would have averted more deaths than found here, and the estimate might appear low. However, in hindsight, given that the fully vaccinated proportion of the population rapidly increased from 30% to >95% from August 2021 to November 2021 (Fig 2B), this must have considerably protected the population from death over the Delta and Omicron periods. Fig 4 makes evident that the second dose death rate was almost zero over the Delta period, a period when a large part of the population had full vaccination. Some 80–90% had their first vaccination and the death rate of those with one dose only was similarly low. In the same period, the booster had only reached a coverage of some 10%. Thus the protection from death of the primary doses was of great importance, and continued into the Omicron wave. This helps us understand why, in a model simulation with no booster, the deaths were found to increase by 53%, but not 10-fold as in Israel (which lacked the same full vaccination coverage; Gavish et al. (2022) [20]). Results from this type of analysis might be overlooked had the analysis been based on a simple SIR model.

### Scenario III

In the complete absence of vaccination, the model predicts an almost immediate major outbreak of Delta, followed by an even more extreme outbreak in the Omicron period in January 2022 (Fig I in S1 File). The predicted but hypothetical Delta outbreak would have reached a maximum of 15,000 cases and maximum of 500 deaths per week in the NSW 50^+^ population. The real Delta outbreak on the other hand had approximately 75 deaths per week. To put this further in perspective, in terms of deaths per capita per week, the no-vaccination scenario would have had a magnitude ~50% of the huge COVID-19 outbreak in unvaccinated Italy’s population during the first wave in early 2020. It is also 25% more deaths per week than the major Omicron January 2022 outbreak (375 deaths per week; see Fig 2A) in NSW.

Altogether over the study period, there would have been an additional 17,760 deaths upon taking into account waning immunity and susceptible depletion. This estimate is a first approximation and should be considered conservative since our linear DIS model of vaccination status-specific death rates (see Part C of the S1 File) fails to capture nonlinear contagion dynamics that would occur after such a large perturbation (i.e., moving everyone to the unvaccinated class). Indirect herd immunity effects of vaccinations also affect the calculation but are not accounted for in the data-driven model. Thus the death rates *r*_*k*_(*t*) could well be under-estimated for the no vaccination scenario (see Limitations and challenges section below), creating further under-estimation of the total deaths, and thereby reinforcing our acknowledgement that the estimate is conservative. In a preliminary work, we find via simulations of the SIR model that for large *R*_0_ (the basic reproduction number) values (eg *R*_0_>5 as in Delta and Omicron) these indirect effects do not have great influence, but for lower values of *R*_0_ they can cause significant underestimates, and create a limitation for this modelling approach. This is similar to the findings of Eichner et al. (2017) [21] and Scutt et al. (2022) [22]. Hence for larger values of *R*_0_ the method of Jia et al. (2023) [11] should provide reasonable estimates of epidemic final size, as we have checked using “mock data” generated by the SIR model. We plan to explore this in more depth in future work.

Finally, we point out that the SIR model could also fail if used to make predictions for Scenario III. For good predictions, the SIR model requires an initial estimate of the susceptible pool and population immunity. But the publicly available vaccination data for NSW is insufficient to make this calculation (including attendant waning), and certainly data for the period before July 2021 is unavailable for the age-group required. For a more detailed explanation of the difficulties in using the compartmental SIR model, see Limitations and improvements for Scenario III in S1 file.

### Aged care

Fig 4B makes clear that the death rates in residential aged care in NSW and VIC were intermediate between those of the unvaccinated and one-dose vaccinated population aged 70^+^ in NSW, which includes both community-living elderly and residents of aged care facilities. Such a higher COVID-19 death rate for aged care residents as compared to the general 70^+^ population, may be due to multiple reasons including a higher proportion of older adults (>85 years) (the 2021 census data [15] indicated that 18% of 70^+^ in NSW were 85^+^, but in residential aged care 59% were 85^+^), a higher proportion of unvaccinated people (on 28 January 2022, about 7% and 9% of aged care residents in NSW and VIC, respectively, had not received any vaccine, compared with only 1.4% of people aged 70^+^ in NSW [17]), and living in dense share-houses with confined space. Policymakers have suggested that "Australia acted decisively to protect vulnerable RACF [Residential Aged Care Facility] residents…. " (Hall et al.) [23]. This included the requirement for all residential aged care workers to be fully vaccinated by 28 June 2021. But despite these major nationwide efforts, the death rates amongst aged care residents in NSW were still considerable and only second to the disproportionately high rates observed in the unvaccinated.

### Limitations and challenges

- Indirect effects: In some periods, the death rates of the unvaccinated *r*_*u*_(*t*) have been determined under conditions of vaccination i.e., when indirect (herd immunity) effects are present. The death rates thus carry an extra component that makes them larger in magnitude than what would be experienced if they were scaled for no vaccination with no indirect effects. This is mostly relevant to the Scenario III, which is the only place we make the “no vaccination” comparison. As a result the predicted deaths averted by vaccination in Scenario III may be under-estimated and conservative. However, the impact of indirect effects are weaker for highly transmissible diseases (*R*_0_>2 as COVID-19; see Eichner et al. (2017) [21] and Scutt et al. (2022) [22]), as we confirm in the aforementioned preliminary work. Using a dynamical model Gavish et al. (2022) [20] found that ∼73% of the reduction in new daily severe COVID-19 cases in the Delta period was due to direct protection, while the rest were due to indirect protection. It appears that for deaths the proportion would likely be more than 73%. Even lower estimates were found for rotavirus (Weideman et al. (2014) [24]).
- Transmission dynamics: The data-driven modelling approach is unable to account for transmission dynamics needed in Scenario III, when contagion dynamics is involved, and thus lacks generality. However, for some scenarios the data-driven model used here is reasonable, and in others provides mortality estimates that we can at least conclude that the total deaths averted are conservative or lower bounds. It also provides an overview of deaths by vaccination status, and the controversial fate of the unvaccinated which is often debated. Limitations of Scenario III are discussed further in Part C of the S1 File.
- Confounding factors: It is important to note that the results obtained in this study may be affected by potentially confounding factors as age and comorbidity. The study was restricted to the single 50^+^ age group. Lack of data availability prevented analysis of finer age groups.
- The more complex version of Scenario III (*DIS* model): The infection fatality rate was guided by literature values and the observed case fatality ratio of Delta and Omicron. A sensitivity analysis was conducted for this parameter (see Part C of the S1 File).

## Conclusion

The analysis of mortality data has demonstrated the role of the unvaccinated subpopulation in driving the COVID-19 outbreaks and the overrepresentation of the unvaccinated amongst COVID-19 deaths. Over the entire study period, the average weekly death rate for the unvaccinated group was 19.8/100,000, compared with 4.7/100,000, 2.6/100,000, and 1.8/100,000 for the one-dose, two-dose, and three-or-more dose groups, respectively. The risk of death for the unvaccinated was 4.2 times, 7.7 times, and 11.2 times higher, respectively.

Through the creation of counterfactual scenarios, and estimated death rates, we have shown that the Australian vaccination roll-out could be viewed as optimal in design presumably because it responded swiftly in real time to changing events such as different evolving variants of SARS-CoV-2 and the timing of their invasions. Our results corroborate that one of the most impressive features of the Australian roll out was the ability to fully vaccinate the eligible population (16^+^) from 10% to 90% in 5 months (from 7 July 2021 to 7 December 2021). Full vaccination coverage of the 50^+^ population in NSW increased from 20% to 93% in 4 months (from July 22, 2021 to November 21, 2021), while the third dose booster coverage increased from 10% to 80% in 3 months (from December 26, 2021 to March 25, 2022).

Compared to the 50^+^ group who had only received two doses since late November 2021 (when Omicron arrived), the booster remained highly effective, associated with an average weekly mortality that was approximately half of the 2-dose group (2.0 vs. 3.7 per 100,000 from late November 2021 to early July 2022). Although the booster was responsible for averting 1,860 [80% PI: 1,290–2,440] deaths (Scenario II), the expectation was that it would avert far more. The late extensive coverage of the two-dose vaccine (which exceeded 93% on November 21, 2021) presumably contributed in preventing further deaths. Many deaths in the extreme January 2022 outbreak were thus averted by the second dose in addition to the booster. With waning, the second dose protection may have reduced by the second half of 2022 after the study period had ended.

Our estimates suggest the entire vaccination campaign (Scenario III) prevented 17,760 [80% PI: 16,610–18,920] deaths during the 48-week study period. The latter figure should be seen as a conservative first approximation, but the best that can be done until more complete data becomes available. Thus, it is important to acknowledge that the vaccination campaign, overall, has had a significant impact by preventing tens of thousands of deaths in NSW, according to a methodology that is characterized as conservative [10,13]. While the data-driven model may appear simplistic, it does give an overview of the big picture of basic epidemiological trends and processes in the vaccination period. It seems that apart from our work, this has not been done because the Australian data is neither publicly available, nor has it been obtainable from request to government bodies. Ultimately, the study helps policymakers better understand the importance of timely vaccination campaigns and raises public awareness of the effectiveness of vaccination in preventing deaths.

## Supporting information

S1 File S1 File containing supporting figures and tables. (DOCX)
