# Supplementary material for: Assessing the impact of Australia’s mass vaccination campaigns over the Delta and Omicron outbreaks
Source: PLoS One. 2024 Apr 16;19(4):e0299844. doi: 10.1371/journal.pone.0299844 (PMC11020690; doi:10.1371/journal.pone.0299844)
Supplement: S1 File — (DOCX) [file pone.0299844.s001.docx]

**Supplementary Information**

**Index**

**Part A. Weekly COVID-19 Deaths in NSW**

**Part B. Vaccination coverage in NSW**

**Part C. Methods and the data-driven model for selected scenarios**

**Part D. Definitions of COVID-19 deaths and vaccination status**

# Part A. Weekly COVID-19 Deaths in NSW

## **Section 1.** Details of weekly COVID-19 deaths in NSW, **aged 50^+^**

From publicly available weekly reports published by NSW Health[1], we obtained the cumulative 2,956 COVID-19-reported 50^+^ deaths with vaccination status (no dose, one dose, two doses, three or more doses) in NSW, from 8 August 2021 to 9 July 2022. Detailed definitions of COVID-19 deaths and vaccination status are provided in Part D. During this period, there are also 219 COVID-19-under-reported deaths (50^+^) without vaccination status recorded in weekly reports published by NSW Health[1], and 320 COVID-19-unreported deaths (50^+^) without vaccination status and specific time distribution recorded in the ‘NSW COVID-19 Related Deaths’ report published by NSW Health[2]. Weekly COVID-19-under-reported deaths were divided according to the distribution of weekly COVID-19-reported deaths by vaccination status. Also, COVID-19-unreported deaths were distributed by week according to the distribution of COVID-19-reported deaths, and then these weekly COVID-19-unreported deaths were divided according to the distribution of weekly COVID-19-reported deaths by vaccination status. Finally, we obtained a total of 3,495 COVID-19 deaths (including reported deaths, under-reported deaths, and unreported deaths). Our study did not include analyses after August 2022 because of the lack of data on the first and second doses of vaccination in the 50^+^ group. We plotted the weekly COVID-19 deaths in NSW, 50^+^ (Fig A). We have also plotted the total COVID-19 deaths in Australia (dark red) aggregated for all age groups. It is clear that the NSW 50^+^ data represent a significant proportion of these deaths in Australia (3,495 of 9,025 deaths). Moreover, these patterns are qualitatively similar to those observed in most other states in Australia.


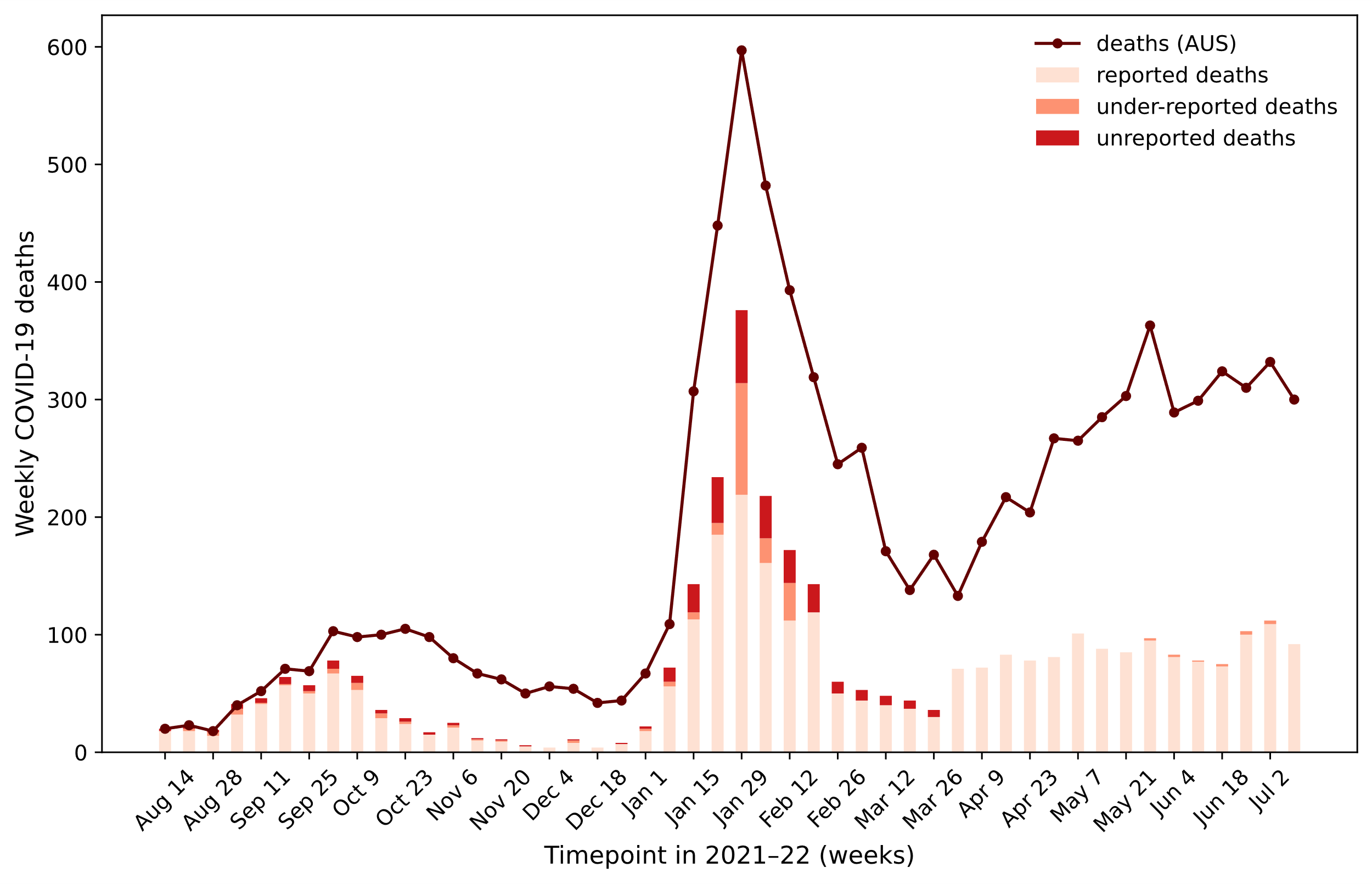


**Fig A. Weekly COVID-19 deaths.** Weekly COVID-19 50^+^ deaths from 8 August 2021 to 9 July 2022 in NSW are shown by bars. Weekly COVID-19 deaths from 8 August 2021 to 9 July 2022 in the whole population of Australia are shown by the dark red line for comparison. Tick is week ending date. The source of the weekly COVID-19 deaths data in Australia is https://ourworldindata.org/.

## **Section 2.** Breakdown of weekly COVID-19 deaths in NSW, **all age groups**

From publicly available weekly reports and the ‘NSW COVID-19 Related Deaths’ report published by NSW Health[1, 2], we obtained a total of 3,613 COVID-19 deaths for all age groups (including 118 deaths aged 50^-^, 506 deaths aged 50-69, 2989 deaths aged 70^+^) from 8 August 2021 to 9 July 2022.

We showed the proportion of COVID-19 deaths in NSW among 50^-^,50-69,70^+^ (Fig B).


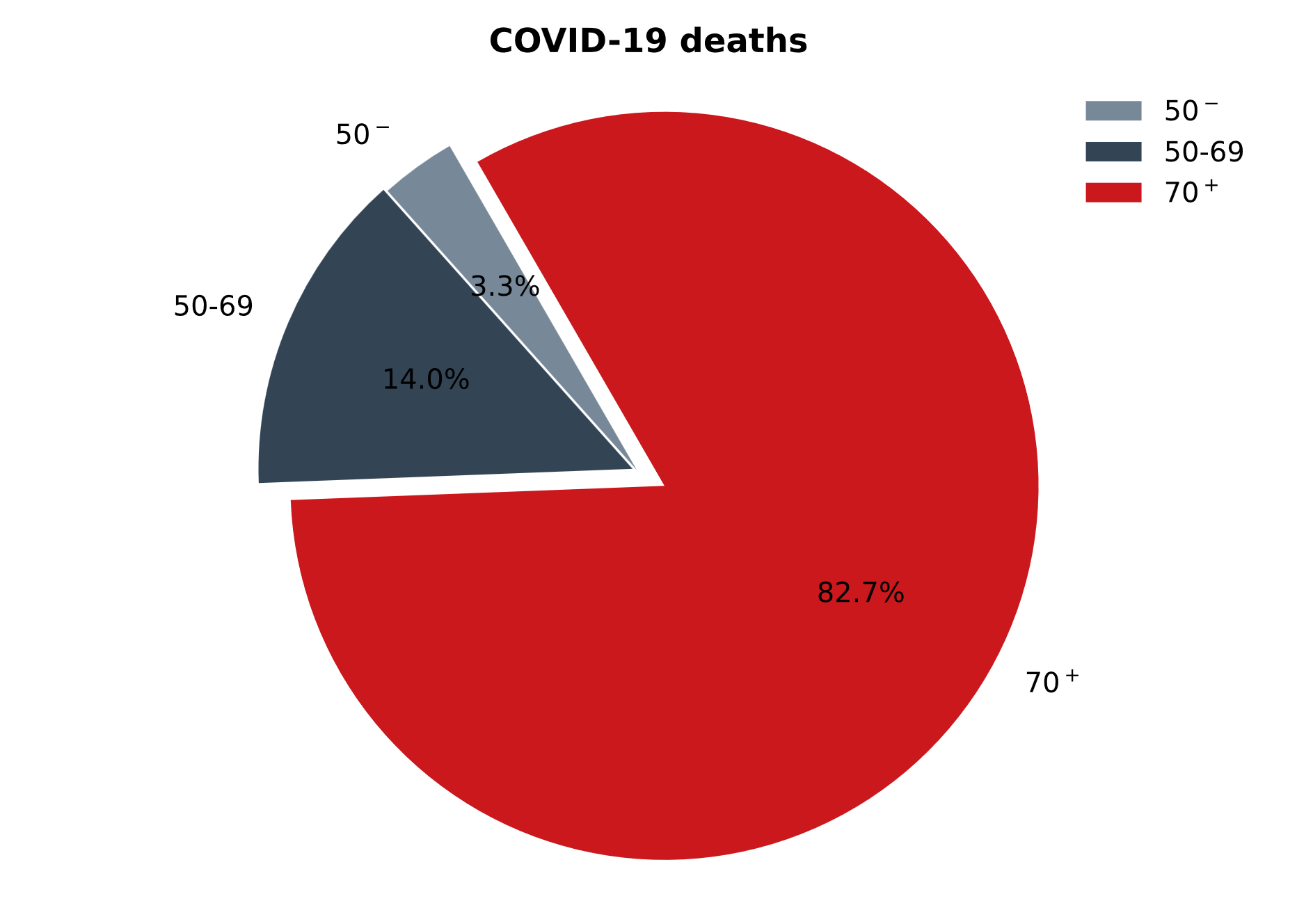


**Fig B. The proportion of COVID-19 deaths.** NSW: The proportion of COVID-19 deaths in NSW among those aged 50^-^, 50-69, 70^+^ from 8 August 2021 to 9 July 2022.

## **Section 3.** Details of weekly COVID-19 deaths in NSW, **aged 70^+^**

From publicly available weekly reports and the ‘NSW COVID-19 Related Deaths’ report published by NSW Health[1, 2], we obtained the cumulative 775 COVID-19-reported deaths with vaccination status (no dose, one dose, two or more doses) in NSW, 70^+^, from 14 November 2021 to 12 February 2022. During this period, there are also 150 COVID-19-under-reported deaths, 70^+^, without vaccination status recorded in weekly reports published by NSW Health[1], and 184 COVID-19-unreported deaths, 70^+^, without vaccination status and specific time distribution recorded in the ‘NSW COVID-19 Related Deaths’ report published by NSW Health[2].

Weekly COVID-19-under-reported deaths were proportionally distributed according to the distribution of weekly COVID-19-reported deaths by vaccination status. Also, COVID-19-unreported deaths were distributed by week according to the distribution of COVID-19-reported deaths, and then these weekly COVID-19-unreported deaths are divided according to the distribution of weekly COVID-19-reported deaths by vaccination status. We obtain a total of 1,109 COVID-19 deaths (including reported deaths, under-reported deaths, and unreported deaths). Our study of those aged 70^+^ was limited to these three months because there was insufficient information to distinguish vaccination status among COVID-19-reported deaths aged 70^+^. Weekly COVID-19 deaths by vaccination status in NSW, 70^+^, are shown in Fig C.


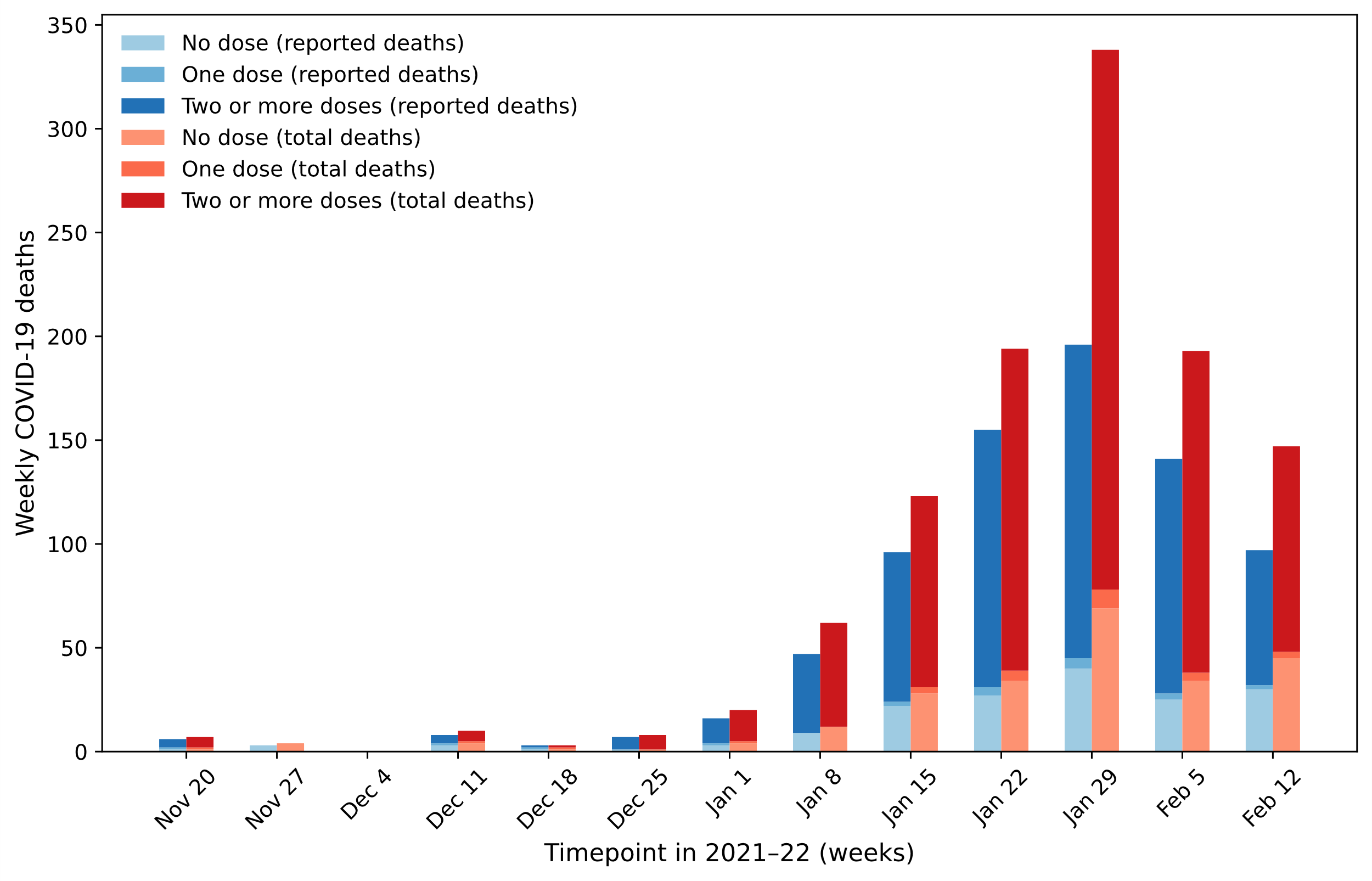


**Fig C. Weekly COVID-19 deaths.** NSW: Weekly COVID-19 deaths (bars in red rows) by vaccination status, 70^+^. Total deaths including reported deaths, under-reported deaths, and unreported deaths. Tick is week ending date.

## **Section 4.** Aged care data in NSW and VIC

On 30 June 2021, there were 63,116 aged care residents in NSW and 49,131 in VIC[3]. The COVID-19 deaths of aged care residents (=299 in NSW; 169 in VIC) were obtained from the Department of Health and Aged Care between 14 November 2021 and 12 February 2022[4]. Axfors et al.[5] found that the Infection Fatality Rate (IFR) was higher in older populations, such as those with a higher proportion of >85 year olds.

# Part B. Vaccination coverage in NSW

## **Section 1.** Vaccination coverage of the 2^nd^ dose of each OECD nation on 22 June 2021

In Fig D, we showed the proportion of people fully vaccinated for countries in the OECD that report the breakdown of doses administered into first and second doses.


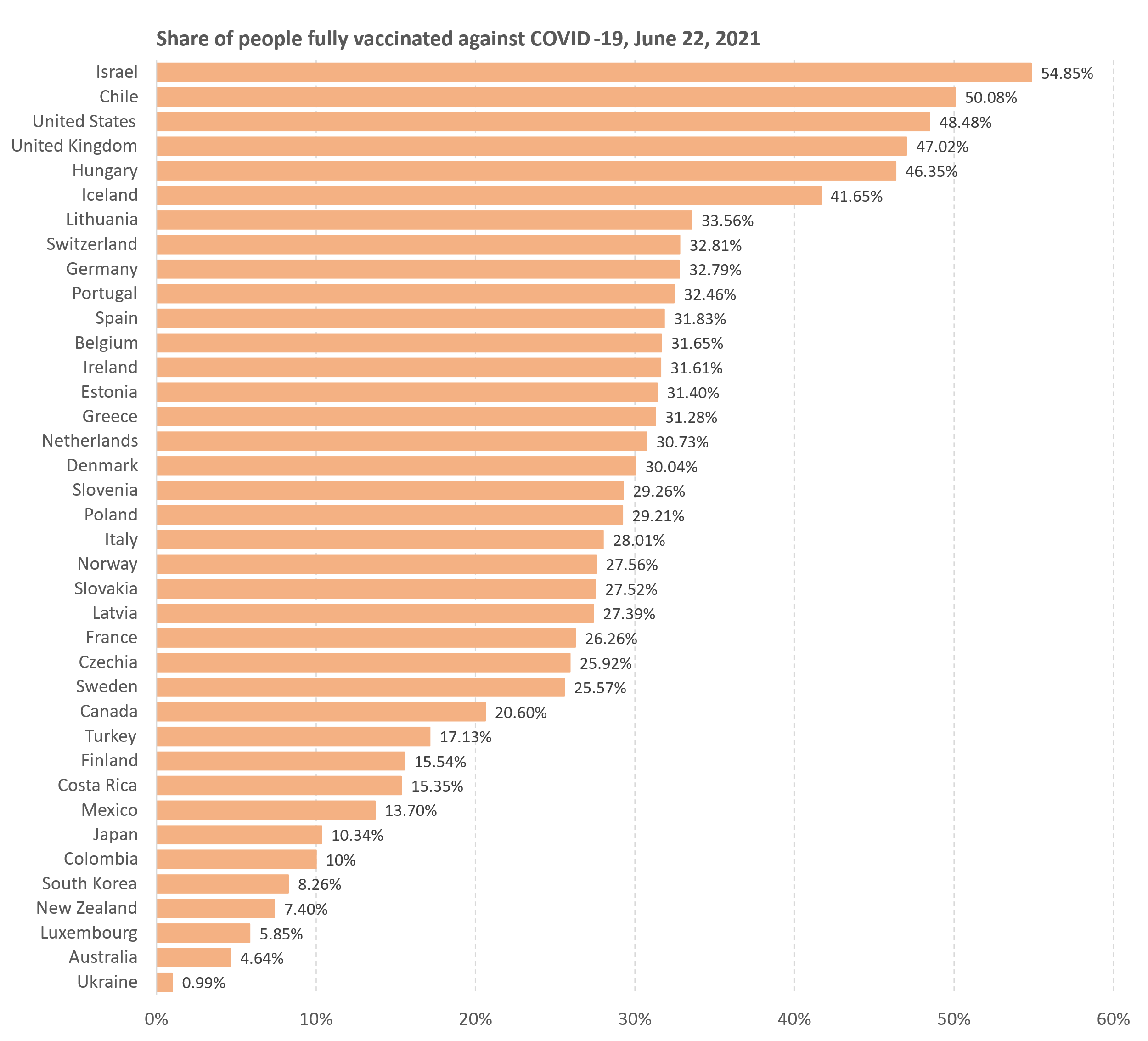


**Fig D. Share of people fully vaccinated against COVID-19, June 22, 2021.** The source of the data is https://ourworldindata.org/.

## **Section 2.** Vaccination coverage of 1^st^ dose and 2^nd^ dose in NSW, 50^+^

The total number of people receiving one and two doses was obtained from the Australian Government’s Department of Health and Aged Care[6]. According to Australian Bureau of Statistics 2021 census data[7], the total number of people aged 50 and over was 2,885,951. We then calculated and plotted the vaccination coverage of 1^st^ dose (purple solid line) and 2^nd^ dose (olive solid line) in Fig E.

## **Section 3.** Vaccination coverage of 3^rd^ dose (booster) in NSW, 50^+^

Fig E depicts our first estimate of the third-dose vaccination coverage among individuals aged 50^+^ in both NSW (blue dashed line) found by shifting the same data for Victoria[8] (grey solid line) by five days. Based on a report[9] which had access to government data, we were able to calculate an independent point (unfortunately only one point) to check our estimate. Namely the proportion of individuals aged 50^+^ in NSW who had received the third dose on February 6, 2022, was 59.03% [represented by the black dot labels as "(Feb 6, 0.5903)"], which is consistent with the estimated value of third-dose vaccination coverage (=0.5913; blue dashed line) on that day in the same age group of NSW.

  We wanted to further test the reliability of our estimates of third dose coverage for people aged 50^+^ in NSW. To do so we made use of data giving the proportion of the Victorian population aged 50^+^ who had received their third dose of vaccine as well as the proportion of those aged 16^+^ who had received their third dose of vaccine[8], over selected dates between November 2021 and February 2022.

If one assumes that the ratio of these two proportions is the same in Victoria as in NSW it is possible to estimate the third dose coverage for people aged 50^+^ in NSW for testing. Namely:

$$\begin{aligned} \frac{VaccCov\left[ NSW50+ \right]\left( t \right)}{VaccCov\left[ NSW16+ \right]\left( t \right)}=\frac{VaccCov\left[ Vic50+ \right]\left( t \right)}{VaccCov\left[ Vic16+ \right]\left( t \right)}.\#\left( S1 \right) \end{aligned}$$

Thus:

$$\begin{aligned} VaccCov\left[ NSW50+ \right]\left( t \right)= \left( \frac{VaccCov\left[ Vic50+ \right]\left( t \right)}{VaccCov\left[ Vic16+ \right]\left( t \right)} \right)VaccCov\left[ NSW16+ \right]\left( t \right).\#\left( S2 \right) \end{aligned}$$

Our second estimate of $VaccCov[NSW50+](t)$ is plotted in Fig E below as seen by the six red dots. The second estimate is very close to our first prediction (blue dashed line) and thus supports our prediction further.

In our study, we didn’t distinguish between the contribution of the third dose (introduced in Australia on 8 November 2021, the formal start of the booster program) and the fourth doses (introduced on 25 March 2022) separately[10, 11]. Instead, we created a ‘three or more doses’ group to combine them together. We also did not distinguish between the efficacy of the AZ vaccine and the Pfizer vaccine.


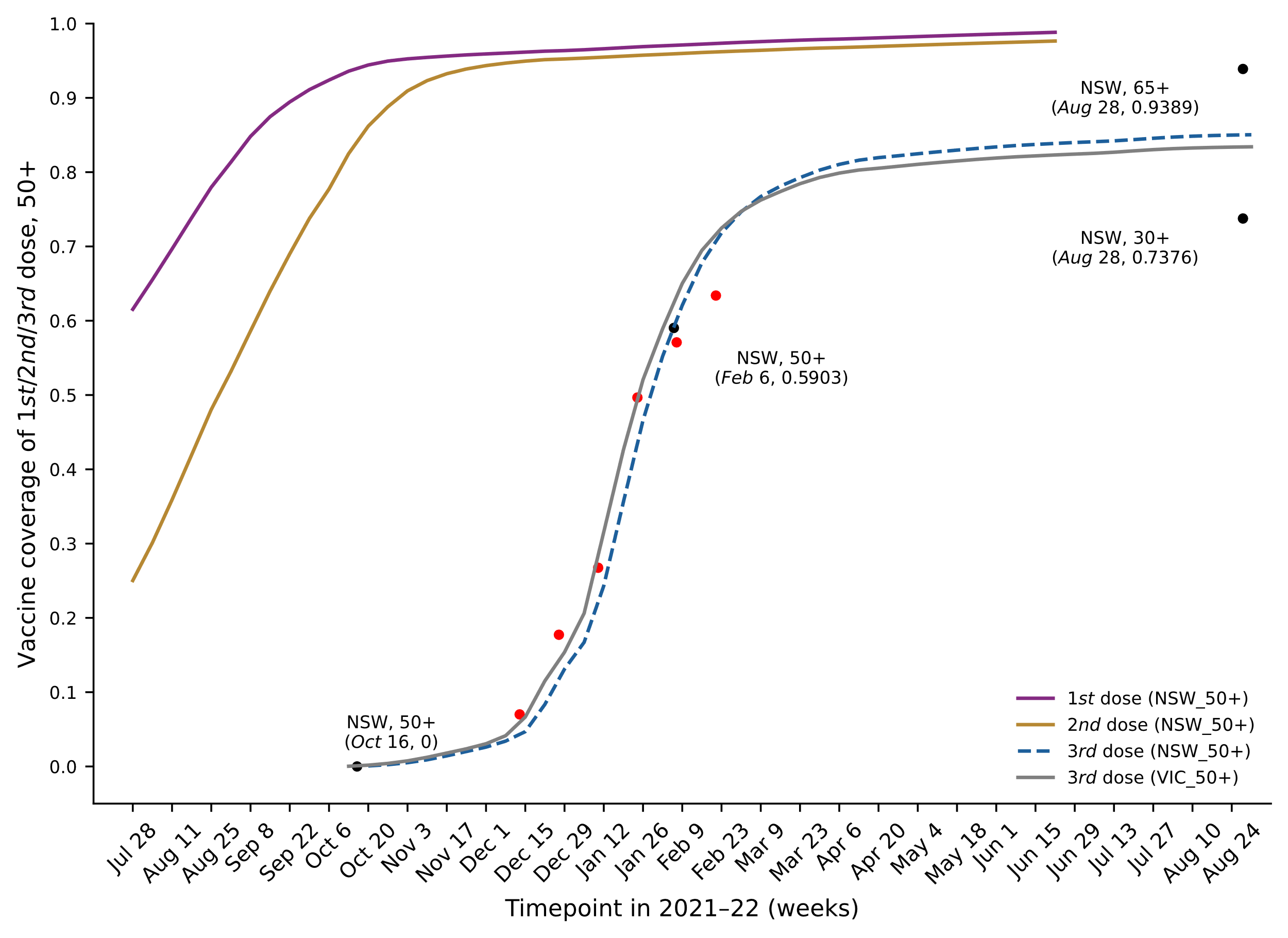


**Fig E. Vaccination coverage of people aged 50^+^ in Victoria and NSW.** Third dose coverage in NSW (blue dashed line) is taken to be similar to VIC (grey line) but shifted 5-days earlier. Corroborated by other points (red, black; see text). Tick is exact date.

# Part C. Methods and the data-driven model for selected scenarios

## **Scenario I.** **How many deaths could have been averted if complete vaccination coverage had been** **achieved early by July 2021?**

We examine how many deaths could have been averted if the entire NSW 50^+^ population had been fully vaccinated as early as July 28, 2021. **This date is two weeks before the study period begins, ensuring that everyone in the population has two *effective* doses by the start of the study period*.***

The updating data-driven model calculates the counterfactual deaths$D_{1}$ as:

$$\begin{aligned} D_{1}=\sum_{t=1}^{48} \left[ {r_{2}\left( t \right)v_{u}\left( t \right)N+r_{2}\left( t \right)v_{1}\left( t \right)N+r}_{2}\left( t \right)v_{2}\left( t \right)N+r_{3+}\left( t \right)v_{3+}\left( t \right)N \right].\#\left( S3 \right) \end{aligned}$$

The terms $r_{2}\left( t \right)v_{2}\left( t \right)N+r_{3+}\left( t \right)v_{3+}\left( t \right)N$ represent those who previously received two and three+ doses and die at a rate $r_{2}\left( t \right)$ and $r_{3+}\left( t \right)$ per week. The terms $r_{2}\left( t \right)v_{u}\left( t \right)N+r_{2}\left( t \right)v_{1}\left( t \right)N$ represent those who were previously unvaccinated, $v_{u}\left( t \right)N$, but now, to accommodate full vaccination, must receive two doses, while similarly, $v_{1}\left( t \right)N$ previously received one dose and now must receive a second dose. All die at a rate $r_{2}\left( t \right)$ per week.

**Incorporating vaccine waning:** However, the effectiveness of vaccines in reducing mortality declines over time as immunity wanes, and this is ignored the above simple method. Thus the total number of deaths, $D_{1}$ calculated using the two-dose death rate (i.e., $r_{2}\left( t \right)$) is an underestimate. Therefore, we have developed an improved method to construct the underlying two-dose death rate $r_{2a}\left( t \right)$ that would have occurred in the absence of any waning. Using this it becomes possible to calculate the estimated deaths with the inclusion of waning.

Grewal et al.[12] found that vaccine effectiveness against Omicron-associated severe outcomes, among subjects aged 70–79 years, decreased from: 84% (95% CI, 57–94%) 7–59 days after a second dose of monovalent mRNA COVID-19 vaccines to 71% (95% CI, 63–78%) after ≥300 days. Thus, the effectiveness of two doses of the vaccine in reducing death rate is assumed to decline by 0.5% per week (about 13% in six months) due to the waning 42 days after vaccination (*t*=5: September 11, 2021).

Based on the number of recorded two- and three-dose vaccinations, we determined each week when those who received only two doses received their second dose. We then calculated the number of people $N_{eff}(t)$ in the "effective" group at week *t* using the time function $e(t)$, i.e., those who are highly protected by two doses of vaccine without waning. The number of people in the "ineffective" group $N_{i\_eff}\left( t \right)$, who no longer have immunity at week *t*, is calculated as $N_{i\_eff}\left( t \right)=v_{2}\left( t \right)N-N_{eff}\left( t \right)$. Note that,

$N_{eff}(t)$: Number of people in the "only two doses" group at week *t* who are in the "effective" group.

$N_{i\_eff}(t)$: Number of people in the "only two doses" group at week *t* who are in the "ineffective" group.

$e(t)$=$\left\{ \begin{aligned} 1, 1\leq t\leq5 \\ 1-0.005\left( t-5 \right), t\geq6 \end{aligned} \right.$

The following equation was used to calculate the weekly death rate of the ‘effective’ group, i.e., $r_{2a}\left( t \right)$. The weekly death rate of ‘ineffective’ group was assumed to be $r_{u}\left( t \right)$.

$r_{2a}\left( t \right)N_{eff}\left( t \right)+r_{u}\left( t \right)N_{i\_eff}(t)=r_{2}\left( t \right)v_{2}\left( t \right)N$, where $r_{2a}\left( t \right)=$max ($r_{2a}\left( t \right)$,0).

In Fig F $r_{2a}\left( t \right)$ is plotted as a function of time and clearly $r_{2a}\left( t \right)$ ${\leq r}_{2}\left( t \right)$ at each time point *t*.

**
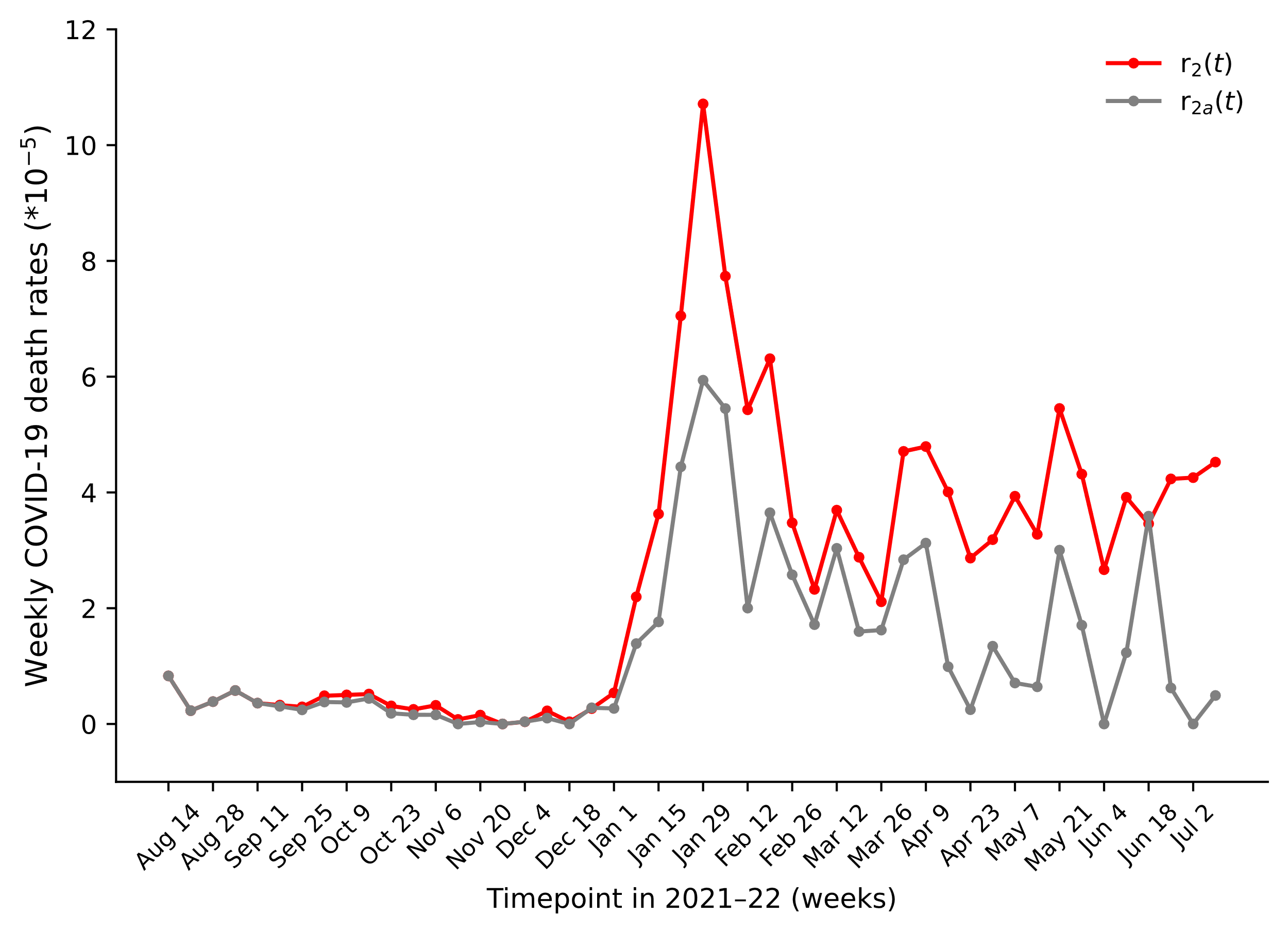
**

**Fig F.** The weekly death rate of two-dose ‘effective’ group $r_{2a}\left( t \right)$ (grey line). Tick is week ending date.

The counterfactual deaths would then be:

$$\begin{aligned} D_{1}^{'}=\sum_{t=1}^{48} \left\{ r_{u}\left( t \right)\left[ 1-v_{3+}\left( t \right) \right]\left[ 1-e\left( t \right) \right]N+r_{2a}\left( t \right)\left[ {1-v}_{3+}\left( t \right) \right]e\left( t \right)N+r_{3+}\left( t \right)v_{3+}\left( t \right)N \right\}.\#\left( S4 \right) \end{aligned}$$

Here, the term $r_{u}\left( t \right)\left[ 1-v_{3+}\left( t \right) \right]\left[ 1-e\left( t \right) \right]N$ represents deaths, at week *t*, due in only two dose subgroups but loses vaccine protection. The term $r_{2a}\left( t \right)\left[ {1-v}_{3+}\left( t \right) \right]e(t)N$ represents deaths, at week *t*, due in only two dose subgroups but with vaccine protection. The term $r_{3+}\left( t \right)v_{3+}\left( t \right)N$ represents deaths, at week *t*, in the booster population.

**The 80% prediction intervals (PIs)** of the counterfactual deaths were calculated based on the uncertainty of the observed weekly death rates, assumed to be binomially distributed, as in Kayano and Nishiura et al.[13] (2022), as follows:

In Eqn.S4, for first term ${T1(t)=r}_{u}\left( t \right)\left[ 1-v_{3+}\left( t \right) \right]\left[ 1-e\left( t \right) \right]N$, we set $n1\left( t \right)= \left[ 1-v_{3+}\left( t \right) \right]\left[ 1-e\left( t \right) \right]N$, ${p1(t)=r}_{u}\left( t \right)$. Then $E\left( T1\left( t \right) \right)\sim B\left( n1\left( t \right),p1\left( t \right) \right)$.

For the second term ${T2(t)=r}_{2a}\left( t \right)\left[ 1-v_{3+}\left( t \right) \right]e\left( t \right)N$, we set $n2(t)= \left[ 1-v_{3+}\left( t \right) \right]e\left( t \right)N$, ${p2(t)=r}_{2a}\left( t \right)$. Then $E\left( T2\left( t \right) \right)\sim B\left( n2\left( t \right),p2\left( t \right) \right)$.

For third term ${T3(t)=r}_{3+}\left( t \right)v_{3+}\left( t \right)N$, we set $n3(t)= v_{3+}\left( t \right)N$, ${p3(t)=r}_{3+}.$

Then $E\left( T3\left( t \right) \right)\sim B\left( n3\left( t \right),p3\left( t \right) \right)$.

Putting this all together: $E\left( D_{1}^{'}\left( t \right) \right)=E\left( T1\left( t \right) \right)+E\left( T2\left( t \right) \right)+E\left( T3\left( t \right) \right)$.

The 80% PI of prevented deaths is the interval between the 10% quantile and the 90% quantile that is derived from 100,000 simulations, assuming the binomial process above. It should be noted that the computed uncertainty bounds do not take into account the structure of the serial dependency and may therefore be conservative.

Finally, in Fig G, we plot the weekly COVID-19 deaths of scenario I (simple) and I (improved) to compare the difference when including waning immunity.


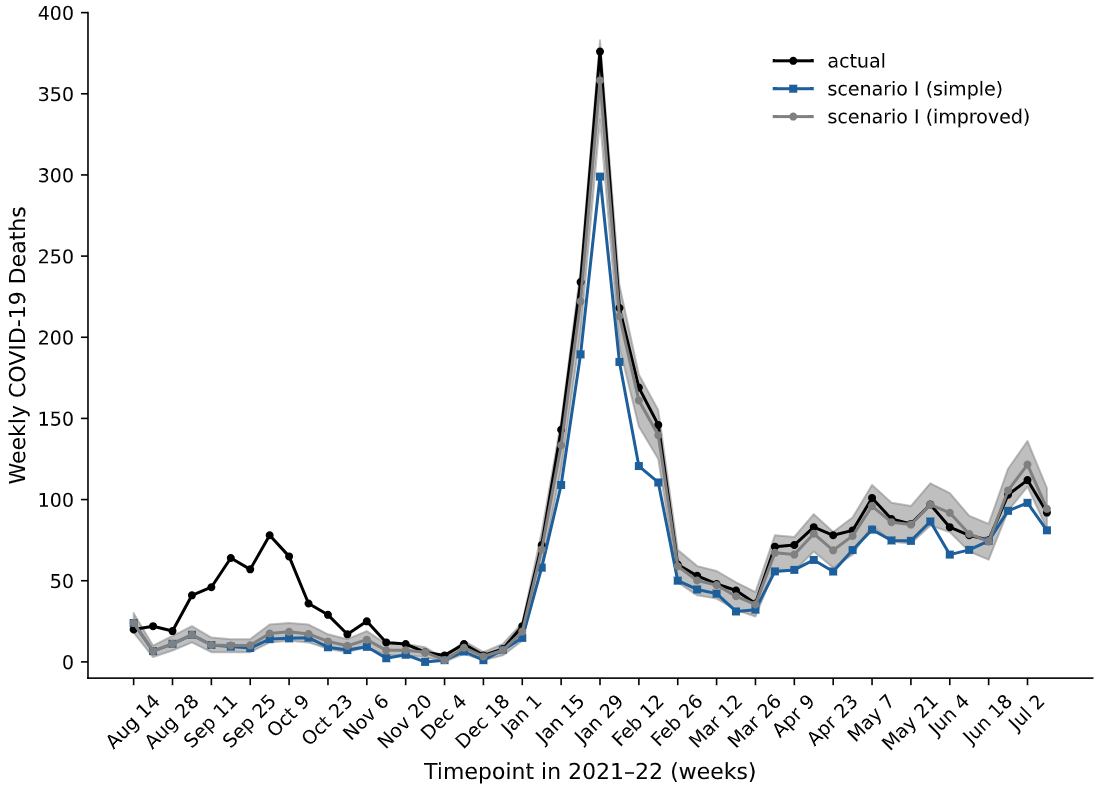


**Fig G.** **Weekly COVID-19 deaths in NSW individuals aged 50^+^.** **Scenario I (simple)** the entire study cohort is fully vaccinated from 28 July 2021 (blue solid); **Scenario I (improved)** as **I (simple)**, but with additional waning immunity (grey solid). The shaded areas are the 80% PIs for the estimate of weekly COVID-19 deaths in Scenario I (improved). Tick is week ending date.

## **Scenario II.** **How many deaths were averted by the booster vaccine?** Equivalently we estimate how many lives would have been lost if Australia failed to provide the booster vaccination, such that no-one in the population was vaccinated with three or more doses?

The counterfactual deaths$D_{2}$ is given by:

$$\begin{aligned} D_{2}=\sum_{t=1}^{48} \left[ {r_{u}\left( t \right)v_{u}\left( t \right)N+r_{1}\left( t \right)v_{1}\left( t \right)N+r}_{2}\left( t \right)v_{2}\left( t \right)N+r_{2}\left( t \right)v_{3+}\left( t \right)N \right].\#\left( S5 \right) \end{aligned}$$

In this counterfactual scenario, those in the ‘three or more doses’ group should return back to the ‘only two doses’ group. Similar to Scenario I (improved), the inclusion of additional waning immunity is necessary for this scenario.

Based on the number of recorded two- and three-dose vaccinations, and the time function $e\left( t \right)$, we determined each week when those who received three or more doses received their second dose. We then calculated the number of people $N_{b\_eff}(t)$ in the "two-dose-effective" group at week *t* using $e(t)$. The number of people in the "two-dose-ineffective" group $N_{b\_i\_eff}\left( t \right)$ at week *t*, is calculated by $N_{b\_i\_eff}\left( t \right)=v_{3+}\left( t \right)N-N_{b\_eff}\left( t \right)$. Note that,

$N_{b\_eff}(t)$: Number of people in the "three or more doses" group at week *t* who are divided into the "two-dose-effective" group.

$N_{b\_i\_eff}(t)$: Number of people in the "three or more doses" group at week *t* who are divided into the "two-dose-ineffective" group.

$e\left( t \right)$, $r_{2a}\left( t \right)$ used in this scenario are the same as those used in Scenario I (improved).

At week *t*, those in the "two-dose-effective" group suffer a death rate $r_{2a}\left( t \right)$, and those in "two-dose-ineffective" group suffer a death rate $r_{u}\left( t \right)$.

The counterfactual deaths would then be:

$$\begin{aligned} D_{2}^{'}=\sum_{t=1}^{48} \left[ {r_{u}\left( t \right)v_{u}\left( t \right)N+r_{1}\left( t \right)v_{1}\left( t \right)N+r}_{2}\left( t \right)v_{2}\left( t \right)N+{r_{2a}\left( t \right)N}_{b\_eff}\left( t \right)+r_{u}\left( t \right)N_{b\_i\_eff}\left( t \right) \right].\#\left( S6 \right) \end{aligned}$$

Similar to Scenario I (improved), the 80% PIs of the counterfactual deaths $D_{2}^{'}(t)$ were calculated based on the uncertainty of the observed weekly death rates.

## **Scenario III and the special DIS model.** **How many deaths would have occurred in the absence of vaccination?**

**Simple data-driven model:**  Based on a simple approximation method, the number of deaths for each week of the 48-week study period is assumed to be the rate at which unvaccinated people with COVID-19 die in that week multiplied by the population size, i.e., $r_{u}\left( t \right)N$.

$$\begin{aligned} D_{3}=\sum_{t=1}^{48} \left[ r_{u}\left( t \right)N \right].\#\left( S7 \right) \end{aligned}$$

This corresponds to Eqn.3 in the main text.

**The improved DIS model:** In general, the simple method used can at best approximate the "big picture". However, when there are large numbers of unvaccinated people, the susceptible pool can change radically over time. A point may be reached where there are not enough susceptible individuals to sustain the dynamics of the infectious disease over a 48-week study period or to model them accurately.

To solve this problem, we used an improved and corrected method sketched here. However, the less technically oriented reader can skip these details. As shown in Fig H, we developed a "D-I-S" model that recursively calculates the number of susceptible individuals, infected individuals, and deaths. In the "D-I-S" model, we use the Infection Fatality Rate (IFR) estimated from literature values and guided by the observed Case Fatality Ratio of Delta and Omicron (see below and Table A). This is used to estimate changes in the susceptible pool which is updated weekly over time. Importantly, the observed weekly death rate $r_{u}\left( t \right)$ of the unvaccinated was taken to be the weekly mortality of the updated susceptible unvaccinated population.

We reviewed the public reports[14] of NSW Health, and calculated that from 16 June to 7 October 2021, the case fatality rate (CFR) of Delta outbreak among the unvaccinated 50^+^ population was 4.45% (=310/6965); Although the number of infection cases may be under-reported, the crude infection fatality rate (IFR) should be approximated by the CFR. Therefore, we take the CFR of the delta variant as the IFR for 50^+^ unvaccinated in NSW. Nyberg and colleagues[15] found that the risk of covid-19 related death was 69% lower in people infected with Omicron than in those with delta. Thus, the IFR of Omicron variant for 50^+^ unvaccinated in NSW was set at 1.38% [=4.45%*(1-69%)].

The values of the parameters for the "D-I-S" model are given in Table A. The values given are taken from the literature.

**Table A. Parameters.**

| **Parameter** | **Value** |
| --- | --- |
| $\rho$: IFR of Delta variant in unvaccinated 50^+^ | 0.0445[14] |
| $\sigma$: IFR of Omicron variant in unvaccinated 50^+^ | 0.0138[14, 15] |
| $\theta$: Effectiveness of previous infection in preventing reinfection with the Delta variant | 1 |
| $\omega$: Effectiveness of previous infection with the Delta variant in preventing reinfection with the Omicron variant | 0.56[16] |
| $\mu$: Effectiveness of post-Omicron infection in preventing reinfection with the Omicron variant | 1 |
| $N$: Number of total 50^+^ population in NSW | 288,5951[7] |


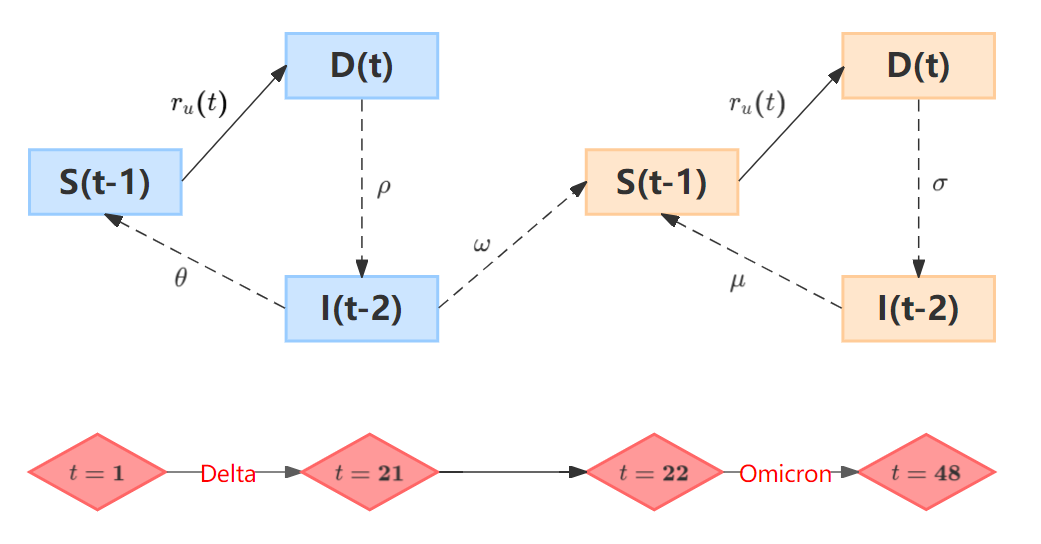


**Fig H. Flows in DIS model over two waves – Delta followed by Omicron.** The LHS summarises the model for the Delta wave having reinfection parameter θ and IFR parameter ρ which at end of week 21 switches to a model of Omicron wave having the same structure but different respective parameters µ and σ. The flowchart of the calculation procedure for D(t), I(t), and S(t) which represent the number of deaths, infections, and susceptible individuals in week *t* respectively. The iterative procedure for calculating D(t), I(t), and S(t) in each week over the study period can be found in Table B. The procedure assumes all COVID-19 deaths up to *t*=21 (i.e., 1 January 2022) are due to the Delta strain (blue model), and all subsequent deaths are due to the Omicron strain (orange model). The susceptibility of the population after the Delta wave is the initial condition for susceptibles in the model for the Omicron wave.

For both the Delta and Omicron strains, we assume 14 days from infection to death[17]. Deaths that occur in the Delta period up to and including week 21 follow the blue model while deaths after week 21 follow the orange Omicron model. The two models have the same structure but different value of parameters.

The whole "D-I-S" model begins by assuming $S\left( 0 \right)=N$. For each week $t$ after the first, the number of susceptible individuals, $S\left( t-1 \right)$, is obtained by subtracting the number of previously infected individuals (protected from reinfection) from the initial susceptible population, $S\left( 0 \right)$. For example, when $23\leq t\leq48$, we have $S\left( t-1 \right)=S\left( 0 \right)-\omega*\sum_{w=1}^{21} I(w-2)-\mu*\sum_{w=22}^{t-1} I\left( w-2 \right)$, where $\omega*\sum_{w=1}^{21} I(w-2)$ represents the number of individuals protected from Omicron infection by antibodies produced by previous infection with the Delta variant, and $\mu*\sum_{w=22}^{t-1} I\left( w-2 \right)$ represents the number of individuals protected from Omicron infection due to antibodies produced by previous infection with the Omicron variant. The specific calculation steps for the iterative procedure are shown in Table B.

**Table B.** $\boldsymbol{I}\left( \boldsymbol{t} \right)\boldsymbol{, S}\left( \boldsymbol{t} \right)\boldsymbol{and}\boldsymbol{D(t)}$ **calculation procedure.**

| $S\left( t-1 \right)=N$  $D\left( t \right)=r_{u}\left( t \right)*S\left( t-1 \right)$  $I\left( t-2 \right)=1/\rho*D\left( t \right)$ | $t=1$ |
| --- | --- |
| $S\left( t-1 \right)=S\left( 0 \right)-$ $\theta*\sum_{q=1}^{t-1} I(q-2)$  $D\left( t \right)=r_{u}\left( t \right)*S(t-1)$  $I\left( t-2 \right)=1/\rho*D\left( t \right)$ | $2\leq t\leq21$ |
| $S\left( t-1 \right)=S\left( 0 \right)-$ $\omega*\sum_{q=1}^{21} I(q-2)$  $D\left( t \right)=r_{u}\left( t \right)*S\left( t-1 \right)$  $I\left( t-2 \right)=1/\sigma*D\left( t \right)$ | $t=22$ |
| $S\left( t-1 \right)=S\left( 0 \right)-$ $\omega*\sum_{w=1}^{21} I(w-2)-\mu*\sum_{q=22}^{t-1} I\left( q-2 \right)$  $D\left( t \right)=r_{u}\left( t \right) S(t-1)$  $I\left( t-2 \right)=1/\sigma*D\left( t \right)$ | $23\leq t\leq48$ |

Finally, in Fig I we show the estimated weekly number of infections, deaths, and the susceptible individuals over the time ($t\in[-1,48]$, July-28, 2021–July-6, 2022) according to the DIS model. The counterfactual deaths would then be: $D_{3}^{'}=\sum_{t=1}^{48} D(t)$.

Similarly, the 80% PIs of the counterfactual deaths $D_{3}^{'}(t)$ were calculated based on the uncertainty of the weekly death rate $r_{u}\left( t \right)$.


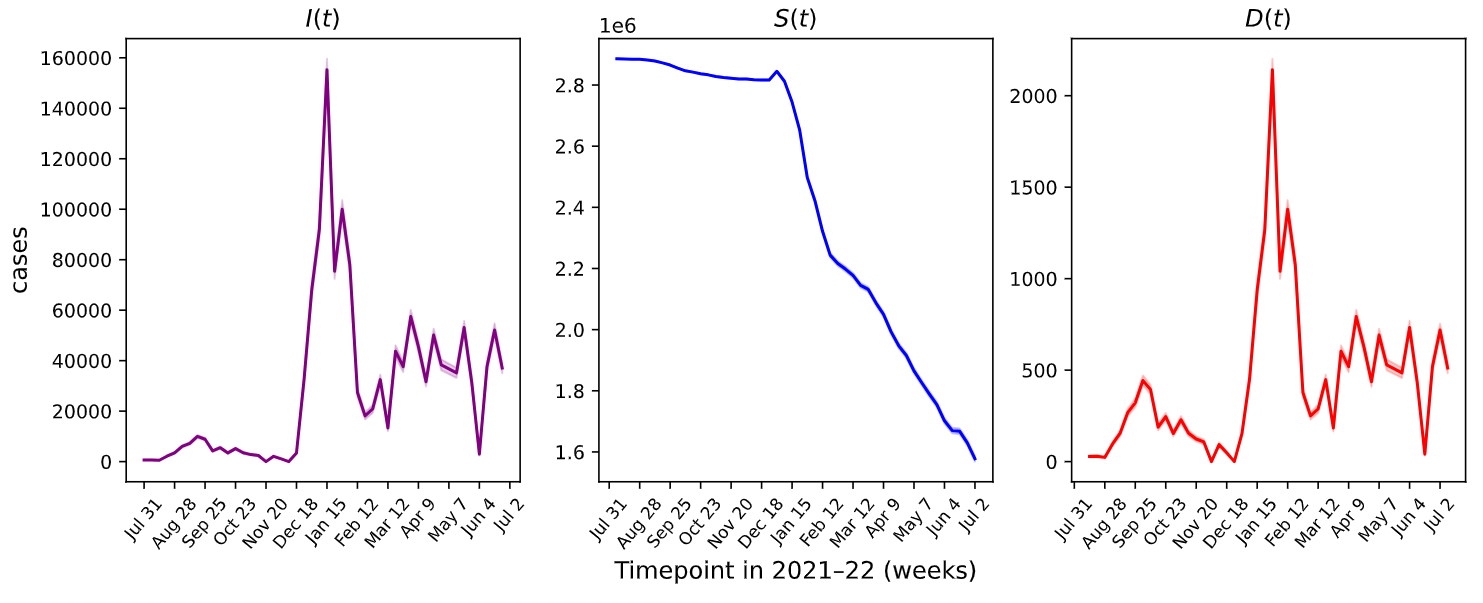


**Fig I. Estimated weekly number of infections, susceptibles, and deaths.** The y-axes show the estimated weekly number of infections, susceptibles, and deaths with 80% PIs in Scenario III. Tick is week ending date.

## **Limitations and improvements for Scenario III.**

**i)** As mentioned with regard to Scenario III, when all vaccination is removed in the simple model, contagion dynamics should set in and the infectives in the population are likely to grow considerably in time and thus qualitatively different to the dynamics of the observed deaths which it is supposed to mirror. Because the simple data-driven model is unable to reflect contagion dynamics in these circumstances, a conservative estimate of deaths is expected. To overcome this limitation, we developed a more suitable DIS-model based on a model of infection and susceptible dynamics using parameter estimates from the literature. The other Scenarios (I&II) are relatively unaffected by this limitation because each can be considered a perturbation of the observed scenario and will be better guided by observed secular trends in mortality.

**ii)** Note that to quantify the mortality rate in the unvaccinated susceptible population, we used the observed mortality rate in unvaccinated individuals $r_{u}\left( t \right)$ as an approximate proxy. However, this slightly underestimates the mortality rate in the unvaccinated susceptible population, resulting in a conservative total number of deaths.

**iii)** As mentioned in the main text, we do not attempt to assess indirect effects. As Jia et al.[18] comment: “The analysis estimated only the effects that could have been achieved by vaccinating unvaccinated persons, reducing their risk of infection and of death if infected, but not the effects of vaccinating these individuals on reducing transmission. A fully dynamic model incorporating these indirect effects would show a different shape for the epidemic curve in a more fully vaccinated population, as earlier vaccination of more individuals delayed infection and potentially changed the epidemic dynamics through shifting patterns of vaccine- and infection-acquired immunity[19]. However, relevant data are lacking to parameterize a model in a way that accurately accounts for prior infection by vaccination status, probability of vaccine breakthrough infection, waning immunity, and compliance with mitigation measures.” Added to these parameters would contact rates, the changing $R_{0}(t)$, reinfection rates, immunity loss and distribution parameters, multiple vaccine parameters (efficacy etc), generation time distribution, time delays etc which are known with little accuracy, and are difficult to infer given the very large number of these key parameters. It is well known that SIRS-type differential equation models cannot provide accurate estimates of deaths (Grad et al. (2012)[20]) without prior knowledge of key parameters. Thus, it is difficult to know which is preferable for studying vaccine campaigns: high quality simulations achievable with a complex dynamical model that is based on parameters some or many of which might not be accurately estimated, versus our data-driven approach that works with measurable mortality rates and provides conservative estimates.

To make things worse, estimates of population susceptibility are required to initiate the simulation, and this depends on unknown previous infections and vaccination history. But the publicly available vaccination data for NSW is insufficient to make this calculation, and certainly publicly available data for the period before July 2021 is unavailable for the age-group required, and attendant waning should be dealt with too.

**iv)** Previous related models have intrinsic limitations that we have attempted to overcome. Jia et al.[18] did not adjust mortality rate as needed to allow for waning immunity. Thus, their conclusions: "that at least 232,000 deaths could have been prevented among unvaccinated adults during the 15 months had they been vaccinated with at least a primary series." are likely to overestimate the effectiveness of the vaccine. Haas et al.[21] similarly estimated the performance of a nationwide vaccination campaign in preventing SARS-CoV-2 infections and deaths in Israel. These works can in some situations lead to errors by not considering waning immunity and susceptible/infection dynamics as mentioned above. The improvements described here should increase the reliability of the results. Due to the lack of more detailed data on vaccination and COVID-19 deaths with age structure in NSW or any State in Australia, we did not perform a more detailed analysis by age.

## **Final results for all scenarios.**

**Table C.** **Estimated counterfactual averted deaths with 95%PI for each scenario.**

| **Type** | **Total estimated deaths (**$\boldsymbol{D}_{\boldsymbol{s}}$**)** | **Averted deaths (**$\boldsymbol{D}_{\boldsymbol{averted}}$**)** |
| --- | --- | --- |
| Actual observed deaths | 3,495 | 0 |
| I (simple). Early achievement of high vaccination coverage | 2,564 | +931 |
| I (improved). Early achievement of high vaccination coverage | 3,056 [95%PI: 2,427-3,730] | +439 [95%PI: -235-1,068] |
| II (simple). No booster campaign | 4,506 | -1,011 |
| II (improved). No booster campaign | 5,354 [95%PI: 4,487-6,270] | -1,859 [95%PI: 992-2,775] |
| III (simple). No vaccination program | 27,363 | -23,868 |
| III (improved). No vaccination program | 21,248 [95%PI: 19,510-23,031] | -17,753 [95%PI: 16,015-19,536] |

## **Sensitivity Analysis (IFR).**

We considered different values for the IFR of the Delta variant in the unvaccinated population aged 50^+^. Note that in this sensitivity analysis the IFR of the Omicron variant changes together with the IFR of the Delta variant. Following Nyberg and colleagues[15], we set the IFR of Omicron to 31% of Delta.

For the baseline, the IFR for Delta and Omicron were set at 0.0445 and 0.0138, respectively.

Varying the IFR value for Delta in the range [0.03-0.05] has only a small effect on the estimated total number of deaths in Scenario III (improved), as shown in the Fig J.


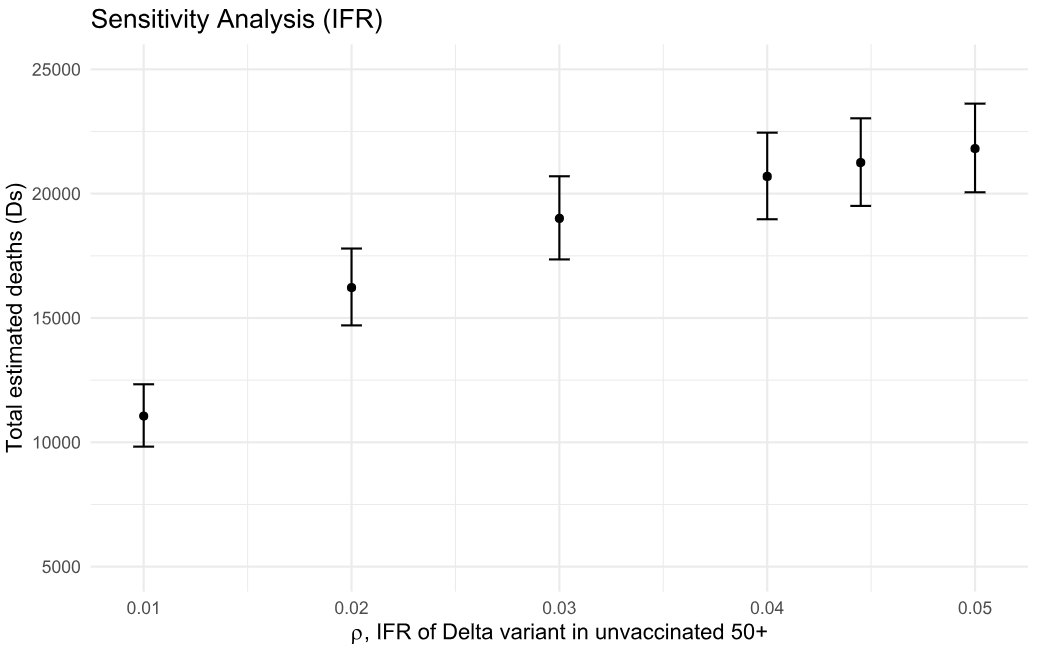


**Fig J.** Sensitivity analysis of the estimated total number of deaths with 95% PI for Scenario III (improved), for different values of the IFR (=0.01, 0.02, 0.03, 0.04, 0.0445, 0.05) of the Delta variant in the unvaccinated population aged 50^+^.

**Incorporating reporting delay for death data:** Not all deaths reported by NSW Health occur in the week in which they are reported, and there may be a delay between when a death occurs and when it is reported to NSW Health. The NSW Health reports suggest that this is sometimes the case. We therefore assume that deaths are reported one week later than they actually occurred, which we implement by shifting vaccination coverage another one week to the right. We recalculate all relevant indicators in this way and obtain the following results in Table D. We find that the effect of the delay (reported in Table D) did not have a considerable impact on the estimated total number of deaths in each scenario (compare with Table C).

**Table D. Estimated counterfactual averted deaths with 95%PI for each scenario considering a delay in reporting death data.**

| **Type** | **Total estimated deaths (**$\boldsymbol{D}_{\boldsymbol{s}}$**)** | **Averted deaths (**$\boldsymbol{D}_{\boldsymbol{averted}}$**)** |
| --- | --- | --- |
| Actual observed deaths | 3,495 | 0 |
| I (simple). Early achievement of high vaccination coverage | 2,579 | +916 |
| I (improved). Early achievement of high vaccination coverage | 3,100 [95%PI: 2,464-3,783] | +395 [95%PI: -288-1,031] |
| II (simple). No booster campaign | 4,276 | -781 |
| II (improved). No booster campaign | 5,035 [95%PI: 4,191-5,924] | -1,540 [95%PI: 696-2,429] |
| III (simple). No vaccination program | 25,892 | -22,397 |
| III (improved). No vaccination program | 20,276 [95%PI: 18,590-22,009] | -16,781 [95%PI: 15,095-18,514] |

# Part D. Definitions of COVID-19 deaths and vaccination status

## **Section 1.** COVID-19 deaths

**COVID-19-reported deaths**: COVID-19-reported deaths are the number of deaths of people diagnosed with COVID-19 reported in weekly reports published by NSW Health[1]. According to NSW Health[1], a COVID-19 death is defined for surveillance purposes as a death in a confirmed COVID-19 case, unless there is a clear alternative cause of death that cannot be related to COVID-19 (e.g., trauma). There should be no period of complete recovery from COVID-19 between illness and death. **COVID-19-under-reported deaths**: COVID-19-under-reported deaths are the difference between the total number of COVID-19 deaths recorded and the number of COVID-19-reported deaths in weekly reports published by NSW Health[1].  **COVID-19-unreported deaths**: An additional 331 COVID-19 related deaths were recorded from January 2020 to March 2022 in ‘NSW COVID-19 Related Deaths’ report published by NSW Health[2].

## **Section 2.** Vaccination status

**Unknown/No dose/One dose/Two doses/Three or more doses**: According to NSW Health[1], vaccination status is determined by matching to Australian Immunisation Register (AIR) data. Name and date of birth need to be an exact match to that recorded in AIR. People with unknown vaccination status were unable to be found in AIR, though may have vaccination details recorded in AIR under a shortened name or different spelling.

**No effective dose**: Cases reported as no effective dose received their first dose of a vaccination course less than 21 days prior to known exposure to COVID-19 or have not received any vaccine dose. Using the phrase “no effective dose” indicates that an insufficient period of time has elapsed to allow for maximal immune response provided by the vaccine. It does not indicate that vaccines are ineffective. **One effective dose**: Cases reported as having one effective dose received their first dose of a two-dose vaccination course at least 21 days prior to known exposure to COVID-19, or received their second dose of a two-dose vaccination course less than 14 days prior to known exposure to COVID-19. **Two effective doses**: Cases reported as having received two effective doses have received their second vaccine dose at least 14 days prior to known exposure to COVID-19 and have not yet received an effective third dose. **Three effective doses**: Cases reported as having three effective doses have had a third dose of COVID-19 vaccine at least 60 days after a valid second dose and 14 days prior to COVID infection. This includes people who are immunocompromised and have had a third primary dose (recommended 2-6 months after second dose), and non-immunocompromised people who have had a booster dose.

# References

1. COVID-19 weekly surveillance reports - Archive. NSW Health. 2022 [cited 2023 Oct 1]. Available from: <https://www.health.nsw.gov.au/Infectious/covid-19/Pages/weekly-reports-archive.aspx>.

2. NSW COVID-19 Related Deaths. NSW Health. 2022 [cited 2023 Oct 1]. Available from: <https://www.health.nsw.gov.au/Infectious/covid-19/Documents/nsw-covid-19-related-deaths-march-2022.pdf>.

3. People using aged care. Australian Institute of Health and Welfare. 2022 [cited 2023 Jan 13]. Available from: <https://www.gen-agedcaredata.gov.au/Topics/People-using-aged-care>.

4. COVID-19 outbreaks in Australian residential aged care facilities. Australian Government’s Department of Health and Aged Care. 2022 [cited 2023 Jan 13]. Available from: <https://www.health.gov.au/resources/collections/covid-19-outbreaks-in-australian-residential-aged-care-facilities>.

5. Axfors C, Ioannidis JP. Infection fatality rate of COVID-19 in community-dwelling elderly populations. Eur J Epidemiol. 2022;37(3):235-49. doi: 10.1007/s10654-022-00853-w.

6. COVID-19 vaccination – vaccination data. Australian Government's Department of Health and Aged Care. 2022 [cited 2023 Mar 13]. Available from: <https://www.health.gov.au/resources/collections/covid-19-vaccination-vaccination-data>.

7. 2021 Census All persons QuickStats. Australian Bureau of Statistics. 2021 [cited 2023 Nov 1]. Available from: <https://abs.gov.au/census/find-census-data/quickstats/2021/POA2100>.

8. Weekly COVID-19 vaccine data. Australian Government and Victorian Government. 2022 [cited 2023 Jan 13]. Available from: <https://www.coronavirus.vic.gov.au/weekly-covid-19-vaccine-data#victorians-receiving-a-covid-19-vaccine-by-age>.

9. Two-thirds of over-70s have booster but uptake ‘disappointing’ among young. The Sydney Morning Herald. 2022 [cited 2023 Jan 13]. Available from: <https://www.smh.com.au/national/nsw/two-thirds-of-over-70s-have-booster-but-uptake-disappointing-among-young-20220202-p59t4k.html>.

10. Start of COVID-19 booster vaccination program. Department of Health and Aged Care. 2021 [cited 2023 Jan 13]. Available from: <https://www.health.gov.au/ministers/the-hon-greg-hunt-mp/media/start-of-covid-19-booster-vaccination-program>.

11. Extra booster dose recommended by ATAGI. Jolyon Attwooll. 2022 [cited 2023 Jan 13]. Available from: <https://www1.racgp.org.au/newsgp/clinical/extra-booster-dose-recommended-by-atagi>.

12. Grewal R, Nguyen L, Buchan SA, Wilson SE, Nasreen S, Austin PC, et al. Effectiveness of mRNA COVID-19 vaccine booster doses against Omicron severe outcomes. Nat Commun. 2023;14(1):1273. doi: 10.1038/s41467-023-36566-1.

13. Kayano T, Sasanami M, Kobayashi T, Ko YK, Otani K, Suzuki M, et al. Number of averted COVID-19 cases and deaths attributable to reduced risk in vaccinated individuals in Japan. Lancet Reg Health West Pac. 2022;28:100571. doi: 10.1016/j.lanwpc.2022.100571.

14. Vaccination among COVID-19 cases in the NSW Delta outbreak Reporting period: 16 June to 7 October 2021. NSW Health. 2021 [cited 2023 Jan 13]. Available from: <https://www.health.nsw.gov.au/Infectious/covid-19/Documents/in-focus/covid-19-vaccination-case-surveillance-051121.pdf>.

15. Nyberg T, Ferguson NM, Nash SG, Webster HH, Flaxman S, Andrews N, et al. Comparative analysis of the risks of hospitalisation and death associated with SARS-CoV-2 omicron (B. 1.1. 529) and delta (B. 1.617. 2) variants in England: a cohort study. Lancet. 2022;399(10332):1303-12. doi: 10.1016/S0140-6736(22)00462-7.

16. Altarawneh HN, Chemaitelly H, Hasan MR, Ayoub HH, Qassim S, AlMukdad S, et al. Protection against the Omicron variant from previous SARS-CoV-2 infection. N Engl J Med. 2022;386(13):1288-90. doi: 10.1056/NEJMc2200133.

17. Marschner IC. Estimating age-specific COVID-19 fatality risk and time to death by comparing population diagnosis and death patterns: Australian data. BMC Med Res Methodol. 2021;21(1):1-10. doi: 10.1186/s12874-021-01314-w.

18. Jia KM, Hanage WP, Lipsitch M, Johnson AG, Amin AB, Ali AR, et al. Estimated preventable COVID-19-associated deaths due to non-vaccination in the United States. Eur J Epidemiol. 2023:1-4. doi: 10.1007/s10654-023-01006-3.

19. Bonvini M, Kennedy EH, Ventura V, Wasserman L. Causal inference for the effect of mobility on COVID-19 deaths. Ann Appl Stat. 2022;16(4):2458-80. doi: 10.1214/22-AOAS1599.

20. Grad YH, Miller JC, Lipsitch M. Cholera modeling: challenges to quantitative analysis and predicting the impact of interventions. Epidemiology (Cambridge, Mass). 2012;23(4):523. doi: 10.1097/EDE.0b013e3182572581.

21. Haas EJ, McLaughlin JM, Khan F, Angulo FJ, Anis E, Lipsitch M, et al. Infections, hospitalisations, and deaths averted via a nationwide vaccination campaign using the Pfizer–BioNTech BNT162b2 mRNA COVID-19 vaccine in Israel: a retrospective surveillance study. Lancet Infect Dis. 2022;22(3):357-66. doi: 10.1016/S1473-3099(21)00566-1.
